# Useful experimental aspects of small-wedge synchrotron crystallography for accurate structure analysis of protein molecules

**DOI:** 10.1107/S2059798324011987

**Published:** 2025-01-01

**Authors:** Kunio Hirata

**Affiliations:** a SR Life Science Instrumentation Team, RIKEN SPring-8 Center, 1-1-1 Kouto, Sayo-cho, Sayo-gun, Hyogo679-5198, Japan; Stanford University, USA

**Keywords:** small-wedge synchrotron crystallography, data collection, protein micro-crystallography, SWSX, synchrotrons

## Abstract

Recent advances in low-emittance synchrotron X-ray technology and highly sensitive photon-counting detectors have revolutionized protein micro-crystallography in structural biology. These developments and improvements to sample-exchange robots and beamline control have paved the way for automated and efficient unattended data collection. This study analyzed protein crystal structures such as type 2 angiotensin II receptor, CNNM/CorC membrane proteins and polyhedral protein crystals using small-wedge synchrotron crystallography (SWSX), which dramatically improves measurement efficiency through automated measurement. We evaluated the data quality using SWSX, focusing on ‘massive data collection’. In this context, ‘massive’ refers to data sets with a multiplicity exceeding 100. The findings could potentially lead to the development of more efficient experimental conditions, such as obtaining high-resolution data using a smaller number of crystals. We have demonstrated that the application of machine learning, a modern key component of data science, to classify data groups is an integral part of the analysis process and may play a crucial role in improving data quality. These results indicate that SWSX is one of the essential candidates for crystal structure analysis methods for difficult-to-analyze samples: it can enable diverse and complex protein functional analysis.

## Abstract 2

This study highlights the efficiency and effectiveness of small-wedge synchrotron crystallography in analyzing protein structures through massive data collection and advanced data-processing techniques, including machine learning, resulting in significant improvements in structural information.

## 1. Introduction

In recent years, low-emittance synchrotron X-ray technology has made it possible to use high-flux microfocus X-ray beams (Evans *et al.*, 2011 ▸; Smith *et al.*, 2012 ▸; Hirata *et al.*, 2013 ▸; Ursby *et al.*, 2020 ▸; Nanao *et al.*, 2022 ▸; Schneider *et al.*, 2022 ▸). This technology has enabled data collection from protein microcrystals. Now that more synchrotron MX beamlines with 5 µm beams are available, the collection of data from crystals of a few micrometres in size has become more accessible.

The advent of highly sensitive photon-counting detectors has also accelerated microcrystal structure analysis (Henrich *et al.*, 2009 ▸; Mueller *et al.*, 2012 ▸). In addition, experimental stations have benefited from continuous improvements in sample-exchange robots and beamline-control systems. Sample-exchange robots of various types and sizes enable the loading and unloading of large volumes of sample pins onto and off the goniometer without human intervention. Automated data-collection systems have recently been implemented at synchrotron MX beamlines worldwide by combining these robots and other beamline instruments (Zander *et al.*, 2015 ▸; Hirata *et al.*, 2019 ▸). Unattended data collection has become almost fully realized. The efficiency of data collection has also improved dramatically, and data accumulation has become extremely fast. Many pipelines have been developed for data processing, and many automated beamlines send the results of data processing to users without human intervention (Yamashita *et al.*, 2018 ▸; Gavira *et al.*, 2020 ▸; Gildea *et al.*, 2022 ▸; Basu, Kaminski *et al.*, 2019 ▸; Schneider *et al.*, 2022 ▸).

In the early stages of data-processing pipeline development, systems were proposed to merge many data sets; for example, to select only one high-resolution data set of higher quality. For this purpose, methods using machine-learning techniques such as hierarchical clustering and genetic algorithms were implemented in the pipeline (Giordano *et al.*, 2012 ▸; Foadi *et al.*, 2013 ▸; Santoni *et al.*, 2017 ▸). In the next stage, several methods were reported to perform analysis of structural polymorphism by grouping large amounts of data in some way rather than simply selecting good-quality data (Nguyen *et al.*, 2022 ▸; Matsuura *et al.*, 2023 ▸). In this case, if it is possible to extract a few more physiologically important polymorphic structures from a large amount of data, it becomes more likely that the efficiency of data collection and the collection of large amounts of data would create a new paradigm.

Even with the development of synchrotron-radiation beamline technology, there are still some samples for which structural analysis is challenging. Using the BL32XU beamline (Hirata *et al.*, 2013 ▸), we have successfully achieved several challenging structural analyses. One of the most commonly used methods is small-wedge synchrotron crystallography (Marin *et al.*, 2020 ▸). This method has been beneficial for obtaining high-resolution data when large numbers of crystals of 5–30 µm in size are available. In particular, it has significantly contributed to the high-resolution structural analysis of membrane proteins crystallized using the *in meso* method (Caffrey, 2003 ▸). Multiple crystals are mounted on a sample holder, such as a crystal loop, and partial data of about 5–10° are collected, assuming that each crystal is randomly oriented. This acquisition is repeated to accumulate small-wedge data sets and merge them with other crystallographically equivalent data sets to obtain complete data.

Recently, a method known as serial femtosecond crystallography (SFX) has become popular for data collection using X-ray free-electron lasers. Inspired by this, serial synchrotron crystallography (SSX) has been developed, advancing structural analysis by collecting one image per crystal at synchrotrons. SSX, like SFX, is particularly suited for room-temperature crystallo­graphy, making it effective for dynamic structural analysis. Time resolved and pump–probe measurements to capture the mechanism of action of protein molecules are expected to become principal serial measurements. SSX achieves high-resolution data collection by capturing single diffraction images and utilizing high-speed data-collection techniques. Various methods have been reported for SSX (Stellato *et al.*, 2014 ▸; Martin-Garcia *et al.*, 2017 ▸; Beyerlein *et al.*, 2017 ▸; Owen *et al.*, 2017 ▸; Hasegawa *et al.*, 2017 ▸; Soares *et al.*, 2022 ▸), including still image measurements where the crystal is not rotated and data are processed using Monte Carlo integration.

Another method, serial synchrotron rotation crystallo­graphy (SSROX), uses a fixed target to slightly rotate the crystal, combining elements of small-wedge and serial measurements. This approach is particularly convenient for fully automated measurements using standard robotics under cryogenic conditions, making it ideal for crystals of micrometre and nanometre sizes with unknown diffraction capabilities. Using SSROX, we achieved 1.8 Å resolution data from 600 nm polyhedral protein crystals with an automated system (Abe *et al.*, 2022 ▸).

Small-wedge synchrotron crystallography (SWSX) is a compelling method for collecting data from microcrystals and has several advantages over SSX with ‘still images’. Compared with ‘still’ measurements in SSX, wedge data provide more comprehensive sampling of reciprocal space per crystal, reducing the total number of crystals needed to achieve complete data sets. Since the evaluation of isomorphism relies on comparing reflection intensities, wedge data facilitate this process by providing more robust sampling of intensities and accurate unit-cell parameter estimations.

Based on the experimental data, we will discuss the advantages and disadvantages of SWSX, covering the region between conventional rotational and serial crystallography with still diffraction. Through several validation experiments, we have studied how to set up the experimental conditions for data collection and processing and how to evaluate the properties and isomorphism of the data. Based on these results, we have organized the information to be useful for any experimental design when performing high-resolution structure and phase determination in SWSX.

## 2. Materials and methods

### 2.1. SWSX on type 2 angiotensin II receptor

#### 2.1.1. Crystal preparation and diffraction data collection

The first sample is a human membrane protein, the type 2 angiotensin II receptor, referred to as AT_2_R. This receptor is the so-called G protein-coupled receptor (GPCR), which is essential in regulating blood pressure. We have reported structural analysis of the protein at 3.2 Å resolution (Asada *et al.*, 2018 ▸). Purification, crystallization and crystal cooling were performed as described in the publication. Diffraction data collection was performed on beamline BL32XU (Hirata *et al.*, 2013 ▸) at SPring-8 using the automated data-collection system *ZOO* (Hirata *et al.*, 2019 ▸). Crystals with sizes of 5–20 µm were mounted on a 600 µm long MicroMount (MiTeGen) using a few to several tens of crystals. Using a 10 × 10 µm beam, partial 2–5° data sets with an oscillation width of 0.2° were collected from each crystal and merged for structural analysis. By collecting data with smaller oscillation ranges, such as 2–5°, we increased the dose per angle, which can enhance the resolution of the data, even though the total dose for collecting each wedge, which was set to 10 MGy, remains the same. This value was calculated using *KUMA*, exploiting *RADDOSE* (Paithankar & Garman, 2010 ▸), based on beam parameters and crystal information (Hirata *et al.*, 2019 ▸). When collecting data from microcrystals with small wedges we used the method described here. Firstly, a two-dimensional raster scan was performed after finding the angle where the crystal loop has the largest area in relation to the X-ray beam. A high-speed detector, EIGER X 9M (Dectris), and the automated diffraction spot finder *SHIKA* were used to specify crystal positions suitable for data collection. Wedge data collection was then performed from goniometer coordinates where more than ten diffraction spots were detected within 5 Å. In the case of 5° data collection, once aligned face-on, ±2.5° of oscillation data were collected for each crystal. Each of the wedge data sets was processed with *DIALS* (version 3.6.1; Winter *et al.*, 2018 ▸).

Hierarchical clustering analysis was performed using *KAMO* to classify the wedge sets using the intensity correlation as a distance function (Yamashita *et al.*, 2018 ▸). Scaling and merging were performed on each of the classified data groups using *XSCALE* (version Jun 30, 2023; Kabsch, 2010 ▸). In the case of *KAMO*, there were three steps for data analysis. The first step is to conduct scaling and merging (referred to as run01) and the second step is to re-scale and re-merge the data sets after eliminating the outlier frames in each wedge set based on the first scaling (run02). Finally, the third step is to re-scale and re-merge the wedge sets after the rejection of anormal wedge sets (run03). Complete data sets resulting from hierarchical clustering, where the completeness and multiplicity of the group data sets were greater than or equal to 95% and 5.0, respectively, were used in the structural analyses described below. In this paper, the results from run03 were utilized.

#### 2.1.2. Structural analyses for comparisons

The 3293 wedge data sets collected were used for hierarchical clustering of intensity correlations. 511 clusters met the completeness and multiplicity criteria described in the previous section, and each was automatically merged with *KAMO* (Yamashita *et al.*, 2018 ▸). For comparison of structural information, molecular replacement was applied to each data set using the PDB model of AT_2_R (PDB entry 5xjm) with *MOLREP* from *CCP*4 (Agirre *et al.*, 2023 ▸). The process was followed by 50 cycles of jelly-body refinement using *REFMAC* with a ridge regression of 0.02 (Murshudov *et al.*, 2011 ▸). The output model was input to *phenix.refine* in the *Phenix* package (Zwart *et al.*, 2008 ▸) to refine atomic positions and isotropic *B* factors without picking water molecules. The resolution limit was determined by detecting the point where CC_1/2_ reaches 50%. We quantified the amount of structural information contained in the data using the ‘information gain’ metric as described by Read *et al.* (2020 ▸). This approach involves calculating the Kullback–Leibler (KL) divergence to assess the contribution of each diffraction measurement to the overall likelihood score in crystallographic analysis. The information was obtained by running *Phaser* with the INFO ON command-line option using the model refined with *phenix.refine.*

### 2.2. The case of the membrane protein CNNM/CorC

#### 2.2.1. Preparing proteins and data collection

CNNM/CorC is a membrane protein that belongs to the Mg^2+^ transporters. The preparation of crystals for data collection has been described by Huang *et al.* (2021 ▸). We used a construct of 162 residues of the protein with Met^121^ replaced with SeMet for initial phasing. Automated data collection was performed on beamline BL32XU at SPring-8 using the automated data-collection system *ZOO*. The wavelength was chosen to be 0.9790 Å to enhance the anomalous signal from Se atoms. Crystals with sizes of 5–20 µm were mounted on a 600 µm LithoLoop (Protein Wave) using a few to several tens of crystals. A rotational data set of 10° was collected from each crystal using a 10 µm (width) × 15 µm (height) beam and merged for structural analysis. Here, the dose for data collection of each wedge was set to 5.0 MGy for phase determination according to previous work (Baba *et al.*, 2021 ▸). Collecting in the SWSX manner, once aligned face-on, ±5° of oscillation data were collected after a 2D raster scan as described in Section 2.1.1.

*KAMO* with *XDS* (version Jun 30, 2023; Kabsch, 2010 ▸) as the back end was used for data processing and *XSCALE* was used for merging and scaling. The hierarchical clustering was performed using *KAMO* with intensity-based correlation as the distance. The scaling and merging process was conducted with *XSCALE* in the *XDS* package, taking into account the anomalous signals. Complete data sets resulting from hierarchical clustering, where the completeness and anomalous multiplicity of the group data sets were greater than or equal to 90% and 3, respectively, were used for the analyses described in the following.

#### 2.2.2. Relationship of structural information to multiplicity

We collected 1342 wedges and 299 merged sets were available for comparison. A resolution limit for each was determined by detecting the point where CC_1/2_ reaches 50%. The initial phase determination of this protein was also performed using merged sets. Determinations of heavy-atom sites, phase calculation and density modification were performed with *SHELXC*/*D*/*E* using each data set with different multiplicity levels (Sheldrick, 2015 ▸). Three macro-cycles of 20 cycles of automatic model building with density modification were applied. The CC_map_ of each set was estimated using the phases from *SHELXE* and the refined structural model from molecular replacement, which is assumed to contain correct phase information. The CC_map_ of each data set was calculated using *phenix.getcc_mtz_pdb* in the *Phenix* package, utilizing the refined model and phase information obtained from *SHELX*. We quantified the amount of information contained in the data using the ‘information gain’ metric in *Phaser* as described in Section 2.1.2.

### 2.3. The case of polyhedra protein crystals

#### 2.3.1. Crystallization and data collection

Polyhedra is a natural crystalline protein assembly of polyhedrin monomer produced in insect cells infected by cypovirus. The protein is a capsid that constitutes the polyhedral virus, the crystal structure of which was first determined at the Swiss Light Source (Coulibaly *et al.*, 2007 ▸). The crystal of the polyhedral protein, referred to as PhC, is often used as a standard sample for performance evaluation of microbeam beamlines because of its tiny solvent content (∼19%) and very high diffracting power as a protein microcrystal. PhC was also used in this paper because of its ease of sample preparation and relatively high isomorphism to investigate how merging more data would affect the statistics and structures that are obtained. Crystallization and crystal-cooling protocols have been reported by Abe *et al.* (2021 ▸). A hundred to several hundred crystals, each of approximately 3–5 µm in size, were mounted in nylon loops (Hampton Research) ranging from 800 to 1000 µm. The density of the crystals was controlled by the amount of cryoprotectant in the suspension, which helped to prevent the crystals from clumping together on the loop. The X-ray wavelength was set to 1 Å. Wedge sets were collected on beamline BL32XU at SPring-8 using the automated data-collection system *ZOO*. As described in Section 2.1.1, data collection was conducted in an SWSX manner.

To compare the amount of structural information obtained from data sets with different wedge sizes but the same total dose, measurements were conducted by varying the wedge sizes. This aimed to investigate how changes in multiplicity and the amount of diffraction signal at the same dose would affect the structural information. Data were collected using wedge sizes of 1°, 5° and 10° at a fixed dose of 10 MGy, meaning that the same number of photons was used for each data set. In the case of 10°, 5° and 1° of data collection, frontal ±5°, ±2.5° and ±0.5° oscillation data were collected, respectively. The beam size and absorbed dose for all wedge data collections were set to 5 × 5 µm and 10 MGy, respectively. With a crystal size of 3–5 µm and an oscillation range of 10° or more, potential misalignment between the crystal and beam, related to the oscillation angle (ω) by [1 − cos(ω)], becomes significant. Therefore, the maximum wedge size was limited to 10° in this experiment.

#### 2.3.2. Data processing and structural analysis

*KAMO* with *DIALS* (version 3.6.1; Winter *et al.*, 2018 ▸) as the back end was used for data processing, and *XSCALE* was used for merging and scaling. PhC has *I*23 crystal symmetry and requires breakage of the indexing ambiguity; *KAMO* automatically detects this requirement, applies selective breeding algorithms (Kabsch, 2014 ▸) to the collected data sets and re-indexes when necessary (Yamashita *et al.*, 2018 ▸). Before merging, isomorphic sets were grouped by hierarchical clustering using intensity correlations among wedge sets as the distance function. Data sets with a completeness of 95% and a multiplicity of 8.0 or greater were selected for subsequent studies. The resolution at which CC_1/2_ decreased to 50% was recorded as the *d*_min_ value of each data set, and its relationship to multiplicity when many wedge sets were merged was evaluated in the same way as for AT_2_R and CNNM/CorC.

Anomalous signals were quantitatively investigated to determine whether the structural information increases as the number of data merges increases. For each merged set, we first performed molecular replacement using a model (PDB entry 2oh6) of the known structure and 30 cycles of jelly-body refinement using *REFMAC* with a ridge regression of 0.02. The output model was input to *phenix.refine* in the *Phenix* package (Zwart *et al.*, 2008 ▸) to refine atomic positions and isotropic *B* factors with picking of water molecules. An anomalous difference Fourier map was calculated and peak heights of the sites containing S atoms were calculated by *ANODE* (Thorn & Sheldrick, 2011 ▸) using the phase information of the refined model. At a wavelength of 1 Å, the *f*′′ value of an S atom corresponds to 0.243 e^−^. Incidentally, the Bijvoet ratio corresponds to approximately 0.30%. The peak heights of the anomalous difference Fourier map in σ, extracted from the *ANODE* output, versus the number of wedge sets in each of merged set is examined. An anomalous peak was only investigated at the S atom near Met124. Additionally, we quantified the amount of information contained in the data using the ‘information gain’ metric as described in Section 2.1.2.

### 2.4. Dose-slicing experiment

#### 2.4.1. Data collection from thermolysin crystal

Thermolysin is a metalloprotease that is found in thermophilic microorganisms and is an enzyme that hydrolyzes peptide bonds containing hydrophobic amino-acid residues. Crystallization and crystal cooling have been described by Hirata *et al.* (2019 ▸). The crystal used for diffraction data collection was approximately 100 × 100 × 800 µm in size. The measurements were performed on beamline BL32XU at SPring-8. Firstly, one irradiation point on the crystal was determined and a 360° 50 kGy data set was collected with an oscillation of 0.1°; this measurement was repeated 100 times. This resulted in a total dose of 5 MGy after data collection. In addition, one 360° data set of 5 MGy was collected from the same irradiation point at 0.1° oscillation (hereafter, this 5 MGy data set will be referred to as the r5.0MGy data). The beam size for all measurements was 10 × 15 µm, and the wavelength was 1 Å. Here, the total absorbed dose for the irradiation point is 10 MGy, which was quantified to perform the comparison as if there were no severe radiation damage. Hot spots of radiation damage and the effect of rotating fresh crystals into the beam are common to all data sets because each data set covers the same rotation range.

#### 2.4.2. Normal data processing

100 data sets of 50 kGy, one for each of the 100 sliced sets, were processed independently with *DIALS*, and the processed reflection files were scaled and merged with *XSCALE* to investigate the intensity statistics of the merged sets. The merged data sets are referred to as the ‘50kGy’ data in the following section.

#### 2.4.3. Synthesizing virtual high-dose data from 50 kGy sets

The 50 kGy thermolysin data were used to synthesize virtual higher dose data (see Supplementary Fig. S1 for details). In data collection, a hundred 50 kGy sliced sets were repeatedly collected, with 3600 frames per set (Section 2.4.1). For example, frame number 1 exists throughout the 100 sets; in all 100 sets, frame number 1 was measured from an identical crystal orientation. Then, summing the exact pixel values of the entire 100 frames of frame number 1, it is possible to synthesize a virtual image with 100 times more photons irradiated. By repeating this process along the frame number, the entire data set of 5 MGy irradiation can be synthesized.

In this way, hypothetical 100 kGy, 250 kGy, 500 kGy, 1.0 MGy and 5.0 MGy sets were synthesized: the 100 kGy data set was synthesized by summing each of the frame number 2 sets and 50 sets were finally prepared. The 250 kGy data were synthesized by summing five sets each and 20 sets were prepared. In the same manner, the 500 kGy, 1.0 MGy and 5.0 MGy sets were synthesized and ten, five and one sets were prepared, respectively.

Data processing was performed for the synthesized data sets. Hereafter, the synthesized data sets will be referred to as the v100kGy, v250kGy, v500kGy, v1.0MGy and v5.0MGy data sets, and each one-sweep data set will be referred to as a sliced set. All sliced sets for each virtual dose were regarded as independent data sets and processed by *DIALS*. *XSCALE* was used to merge the resulting reflection files of sliced data sets from *DIALS*. Specifically, 50, 20, ten, five and one reflection files were obtained for the v100kGy, v250kGy, v500kGy, v1.0MGy and v5.0MGy data sets, respectively, and all of them were merged for each virtual dose amount. This study allowed us to compare the effect of the diffraction signal enhancement obtained when the number of incident photons was increased. The total number of photons for each synthesized sets is identical.

#### 2.4.4. Amount of structure information

A resolution limit for each data set was determined by detecting the point where CC_1/2_ decreases to 50%. Molecular replacement was applied to each data set using the PDB model of thermolysin (PDB entry 1kei) with *MOLREP* in *CCP*4. The process was followed by refinement using 50 cycles of jelly-body refinement in *REFMAC* with a ridge regression of 0.02. The output model was input to *phenix.refine* to refine atomic positions and isotropic *B* factors with picking of water molecules. Anomalous difference Fourier maps were synthesized using the phase information from the structural refinement, and the peak heights of the zinc ion positions were calculated and used to compare. These processes were performed using *SHELXC* and *ANODE*. The peak heights of the anomalous difference Fourier maps of zinc ions and the *R*_free_/*R* factors were used to compare the amount of structural information.

## 3. Results and discussion

### 3.1. Case of angiotensin II receptor (type 2)

**Figure 1:**
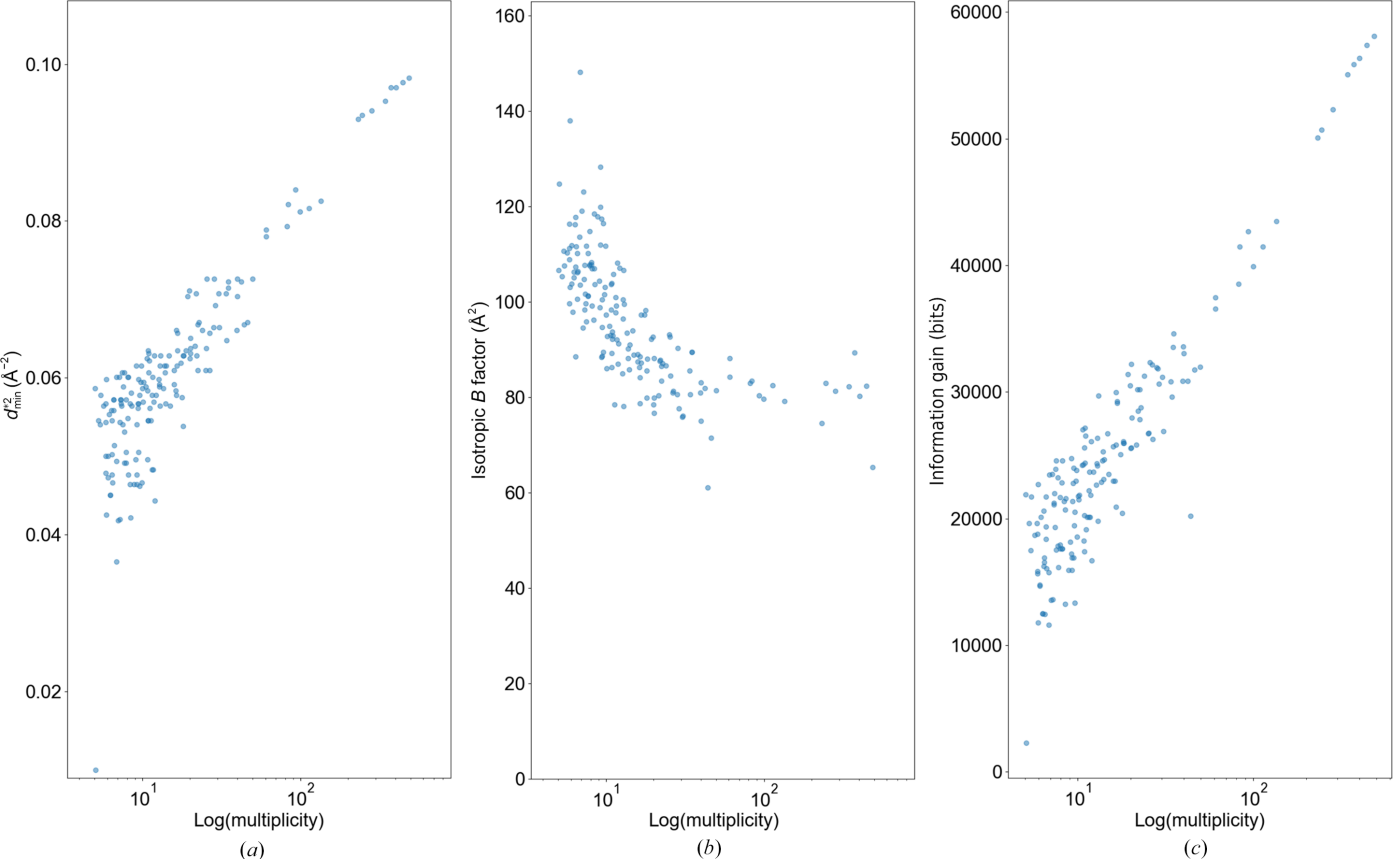
Statistics of AT_2_R merged sets. The plots show (*a*) 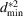, (*b*) isotropic *B* factor and (*c*) information gain from *Phaser* with logarithm of multiplicity on the horizontal axis. Each point represents a merged set.

Fig. 1 ▸ is a plot comparing 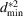, the isotropic temperature factor after refinement and ‘information gain’ calculated with *Phaser* for the AT_2_R data, versus the logarithm of the multiplicity on the horizontal axis. Fig. 1 ▸(*a*) shows quantitative evidence: the resolution limit improves as the number of merged data sets increases. The resolution limit is improved even though the individual wedge sets show lower resolution (data not shown). A similar relationship between the incident number of photons and 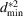 has been reported (Yamamoto *et al.*, 2017 ▸; Winter *et al.*, 2019 ▸) and follows the Wilson plot relationship. The number of data sets can be regarded as the total number of photons for data collection. Fig. 1 ▸(*b*) shows that using redundant data tends to reduce the overall isotropic *B* factor obtained from the refinement results. A smaller *B* factor indicates more accurately determined atomic positions. Considering that *d*_min_ ranges from 3.2 to 3.5 Å and that the *R*_free_ factors are around 30–32% for these data sets, increasing multiplicity suggests that more structural information is included in the final data.

**Figure 2:**
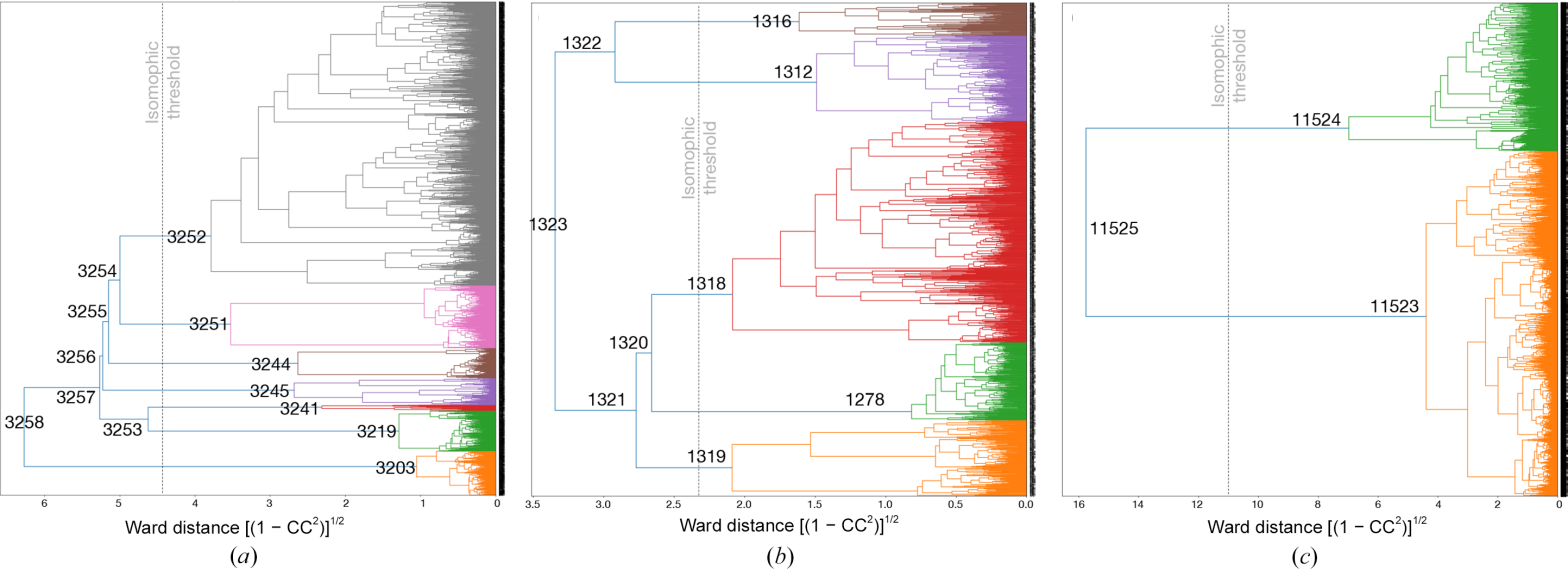
Dendrogram of intensity-based hierarchical clustering of three samples: (*a*) AT_2_R, (*b*) CNNM/CorC and (*c*) PhC. The Ward distance of the clustering is calculated with (1 − CC_2_)^1/2^ as a distance matrix, where CC is a correlation coefficient between each wedge set. The gray dotted line in each plot shows the ‘isomorphic threshold’ calculated by multiplying the maximum Ward distance on the dendrogram. Representative clusters mentioned in the text have IDs labeled at the branching points.

We examined the dendrogram from hierarchical clustering of this sample using intensity correlations among wedge sets as the distance function (Fig. 2 ▸*a*). The dotted line in Fig. 2 ▸(*a*) indicates the ‘isomorphic threshold’ proposed in our previous paper. This threshold is estimated by multiplying the maximum Ward distance in the dendrogram by 0.7. We suggest that cluster nodes with Ward distances below this threshold may contain polymorphic, non-isomorphic, structures (Matsuura *et al.*, 2023 ▸). Clusters 3203, 3219, 3241, 3244, 3245, 3251 and 3252 were identified as potentially polymorphic (hereafter referred to with a ‘C’ prefix; Table 1 ▸ and Supplementary Table S1). After refinement, electron-density maps were compared, but only C3252 had data with a resolution higher than 3.5 Å, making it challenging to detect structural differences. Due to the minimal differences observed in these maps across the seven data sets, merging additional data was considered to be a viable strategy to enhance *d*_min_. For example, merging C3251 and C3252 to form C3254 increased the multiplicity and improved the resolution, without significant issues in the refinement *R* factors. Merging up to the largest data set, C3258, was feasible without adversely affecting refinement quality.

Fig. 1 ▸(*c*) shows the relationship between the information gain calculated by *Phaser* and multiplicity. It shows similar behaviors to those observed in Fig. 1 ▸(*a*), showing that these have a very good correlation. An increase in the information gain calculated by *Phaser* indicates an increase in structural information. This demonstrates that information gain is a good indicator for evaluating the amount of structural information. Collectively, these results suggest that the improvement in *d*_min_ not only represents an apparent value but also indicates a genuine increase in structural information.

### 3.2. The case of CNNM/CorC

**Figure 3:**
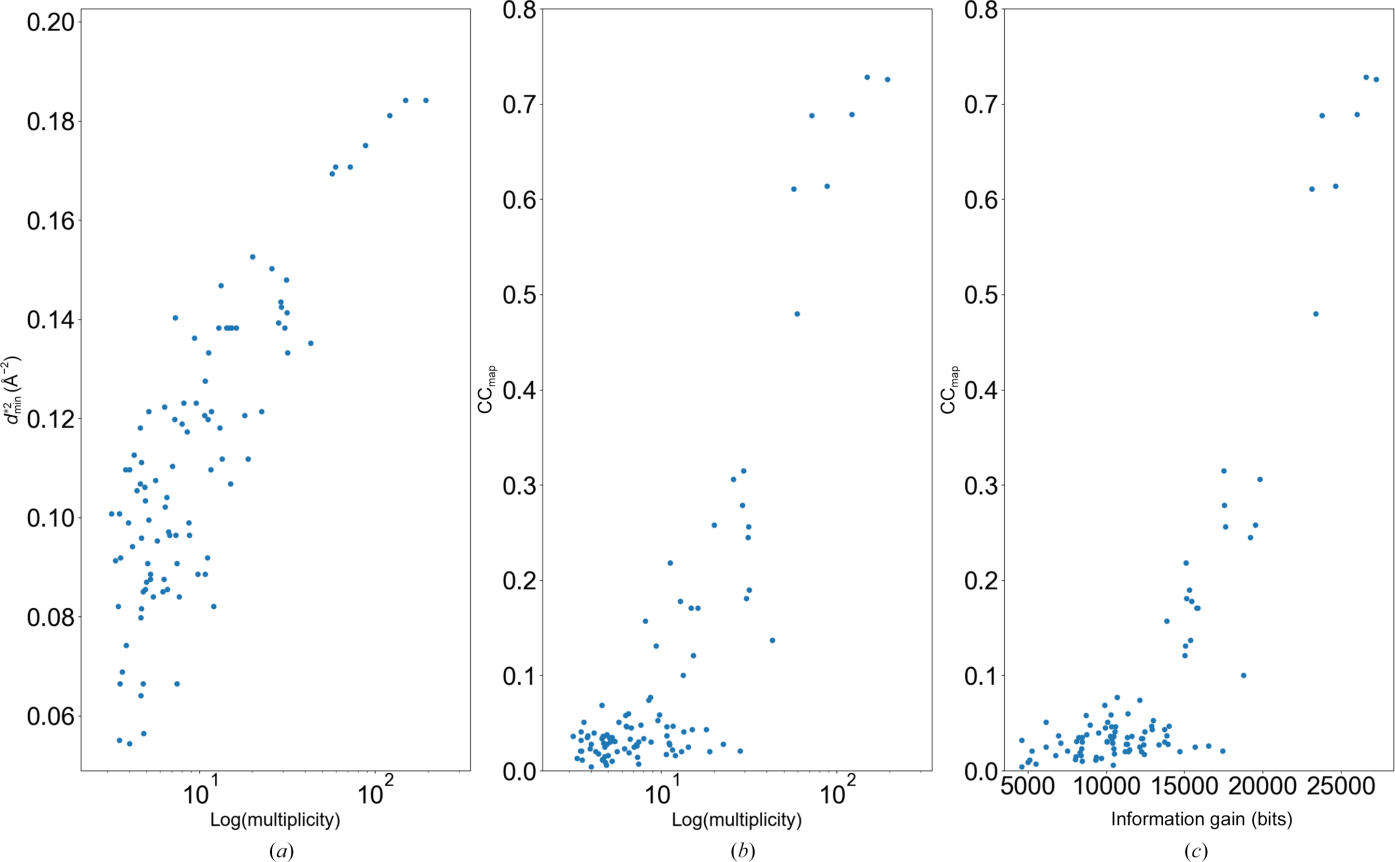
Statistics of CNNM/CorC data sets. The plots show (*a*) 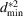, (*b*) CC_map_ and (*c*) the relationship between CC_map_ and information gain from *Phaser*. The horizontal axis in (*a*) and (*b*) is the logarithm of multiplicity, and that in (*c*) is information gain. Each point represents the merged set.

The objective of our CNNM/CorC experiment is to determine the phase of this crystal structure accurately. Fig. 3 ▸(*a*) shows the results of our study, highlighting the relationship between the number of merged data sets and the improvement in *d*_min_. As with AT_2_R, *d*_min_ improves with an increase in the number of merged data sets, showing a clear correlation. Additionally, the graph of CC_map_ versus the number of merged data sets demonstrates that increasing the number of merged data sets has a beneficial effect on accurate phase determination (Fig. 3 ▸*b*). As the number of incident photons and the multiplicity increase, the CC_map_ value improves, directly impacting phase determination. For this sample, a multiplicity of at least 20–30 was required to achieve successful phase determination, defined as having a CC_map_ > 50%. We also investigated the relationship between ‘information gain’ from *Phaser* and CC_map_. The results showed that higher information gain corresponds to higher CC_map_ values (Fig. 3 ▸*c*). From this result, it was evident that information gain effectively represents the amount of structural information contained in the data.

When examining structure refinement, as with AT2R, we categorized the merged data sets potentially containing polymorphic structures based on the isomorphic threshold. Five data sets, C1278, C1312, C1316, C1318 and C1319, were identified as potentially containing polymorphic structures (Fig. 2 ▸*b*). Statistics for each data set are summarized in Table 2 ▸ and Supplementary Table S2. *F*_o_ − *F*_o_ maps were calculated and compared, excluding C1316. However, no structural polymorphism was identified at the current resolution and phase quality. Representative data sets at the branching points of the dendrogram were used for structural refinement, and the resulting *R* values were plotted against d*^2^ (Supplementary Fig. S2). Among these, C1318 yielded the lowest *R*_work_ up to 2.45 Å resolution. Successive merging in the dendrogram increased the multiplicity and improved the resolution. As multiplicity increased with each merging, the resolution limit and the CC_map_ for phase determination improved, while the overall *R* factor worsened. The final data set, C1323, which combines C1322 and C1321, shows that the *R*_work_ of C1321 falls in a range intermediate between those of C1318 and C1321 (Supplementary Fig. S2). The CC_map_ of C1323 showed no significant improvement over C1321, but the number of residues built by *SHELXE* remained the same (142 out of 161), suggesting that both data sets are likely to be valid for phasing.

Merging data sets accumulates signals, improving *d*_min_ and phase determination, but can increase *R* factors at higher resolution (for example C1321 and C1323; Supplementary Fig. S2, Table 2 ▸). It is crucial to evaluate these factors carefully to determine whether the data set fits the purpose of the structural analysis and to select the final cluster accordingly. For this sample, it would be possible to choose C1318, which had the lowest refinement *R* factor up to the higher resolution range, or C1321, which offered higher resolution and could be utilized for phase determination. Results clearly shows that higher multiplicity facilitates phase determination. Previous studies on S-SAD have shown that higher multiplicity improves data accuracy and phase determination (Rose *et al.*, 2015 ▸; Liu *et al.*, 2011 ▸, 2013 ▸; Basu, Olieric *et al.*, 2019 ▸).

Hierarchical clustering and similar data-set grouping methods are particularly important when performing large-scale data collection and merging (Foadi *et al.*, 2013 ▸; Santoni *et al.*, 2017 ▸; Nguyen *et al.*, 2022 ▸). Utilizing such methods to narrow down a large number of data sets to a small subset of isomorphous data enables the more effective selection of data sets suited to the research objectives.

Particularly in phase determination using LCP crystals of membrane proteins, ring diffraction patterns from ordered lipids often appear around 4.3–4.5 Å, making the accurate integration of diffraction intensities in this region challenging. We believe that this is one of the reasons why, in our previous studies, SAD phase determination became difficult when the Bijvoet ratio was not very high. Based on our experience, soaking crystals with heavy atoms such as mercury was necessary for structure determination in such cases (Kato *et al.*, 2012 ▸; Nishizawa *et al.*, 2013 ▸; Kumazaki *et al.*, 2014 ▸). The present results indicate that increasing the multiplicity significantly enhances the potential for structure determination, especially for membrane proteins and proteins with low Bijvoet ratios. Additionally, in challenging phase-determination cases, collecting a large number of homogeneous crystals could allow successful phase determination without substituting heavy atoms.

### 3.3. PhC results

#### 3.3.1. PhC data comparison of 10° wedge sets

A major question arises: as multiplicity increases, does the amount of structural information, as shown in the previous section, continue to increase indefinitely? To investigate this, a large number of wedge sets were prepared from uniformly produced PhC.

The dendrogram in Fig. 2 ▸(*c*) splits the data into two nodes: C11523 and C11524. The CC between the merged data sets of C11523 and C11524 is 0.685, indicating low isomorphism. The *R*_free_ (median) was 19.6% for cluster C11523 (919 grouped data sets at the split node) and 26.1% for cluster C11524 (361 grouped data sets at the split node), with both clusters filtered for *d*_min_ < 2.0 Å before comparison. Statistics showed that C11524 wedge data sets consistently had a lower resolution limit than those in C11523 based on CC_1/2_. In the lowest resolution shell, 5.8% of wedge data sets in C11523 and 65% in C11524 had CC_1/2_ below 90%, indicating poorer quality in C11524. The data set with the lowest refinement *R* factor, C11459, which belonged to one of the subclusters of C11524, had an *R* factor of 17.5%, a *d*_min_ of 1.27 Å and an inner-shell CC_1/2_ of 99.7%, but an *R*_sym_ for the lowest angles (25–2.74 Å) of 42.2% (*R*_p.i.m._ was 2.0%). Further analysis showed that ∼50% of the wedge data sets in C11524 had completeness values roughly half of those in the high-quality data. These differences are likely to stem from variations in crystal diffracting power, alignment quality or spatial overlap during data collection. These results support the clustering result that grouped low-quality data into C11524 and high-quality data into C11523. Here, hierarchical clustering, rather than polymorphism detection, improved data merging by grouping data sets of varying quality. Although *R*_p.i.m._ improved with multiplicity, the C11524 data were clearly of poor accuracy.

**Figure 4:**
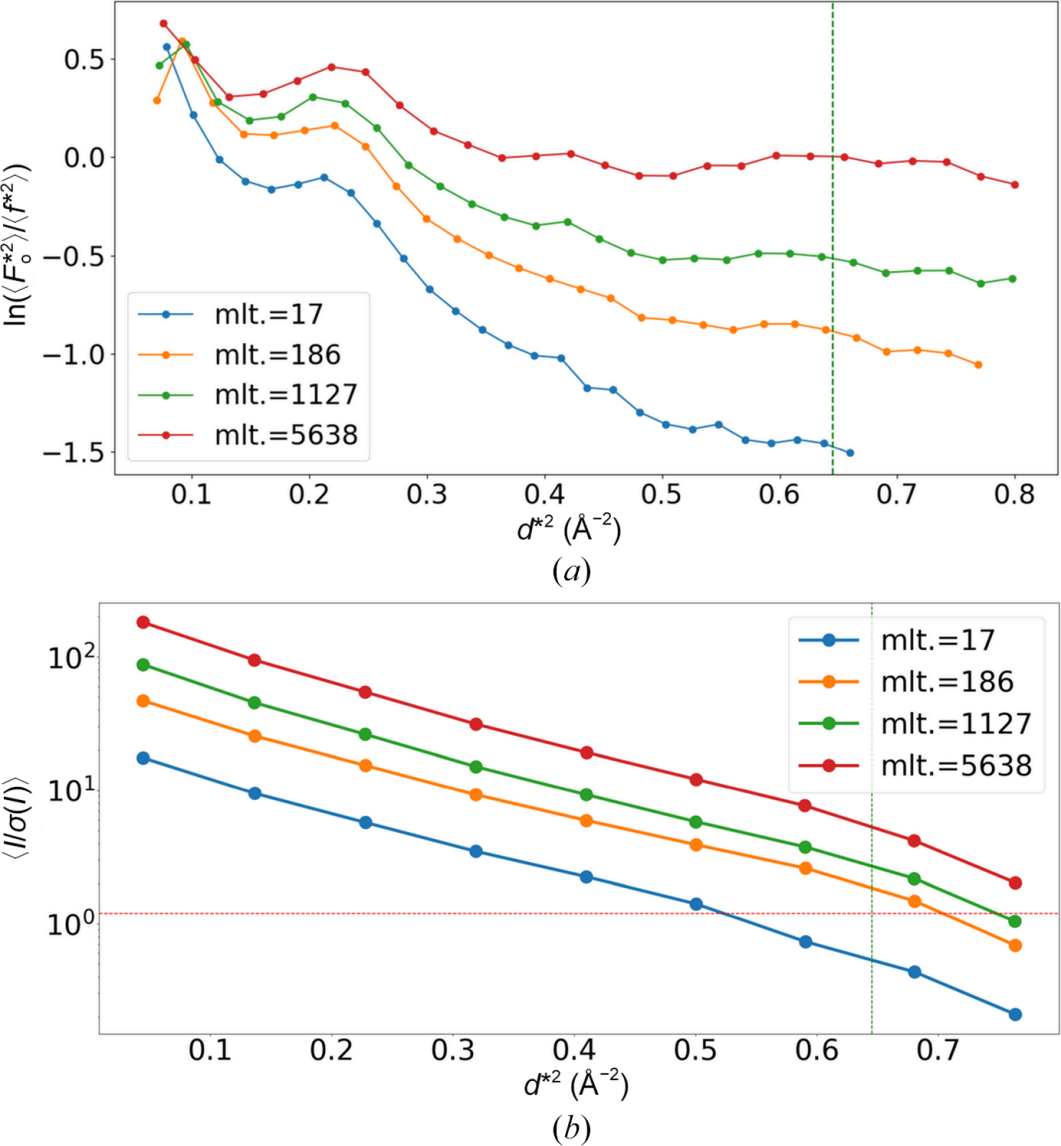
Intensity plot of subclusters of C11523. (*a*) Wilson plot of selected subcluster data sets of C11523. The ‘mlt’ values in the legend represent the multiplicity of the data set. (*b*) 〈*I*/σ(*I*)〉 plot of subcluster data sets in C11523. The *I* and σ(*I*) values were extracted from reflection files obtained from *XSCALE*. The green vertical lines shown in both plots indicate the resolution at the half corner of the detector, 1.25 Å. The horizontal red line in (*b*) represents 〈*I*/σ(*I*)〉 = 2.0.

**Figure 5:**
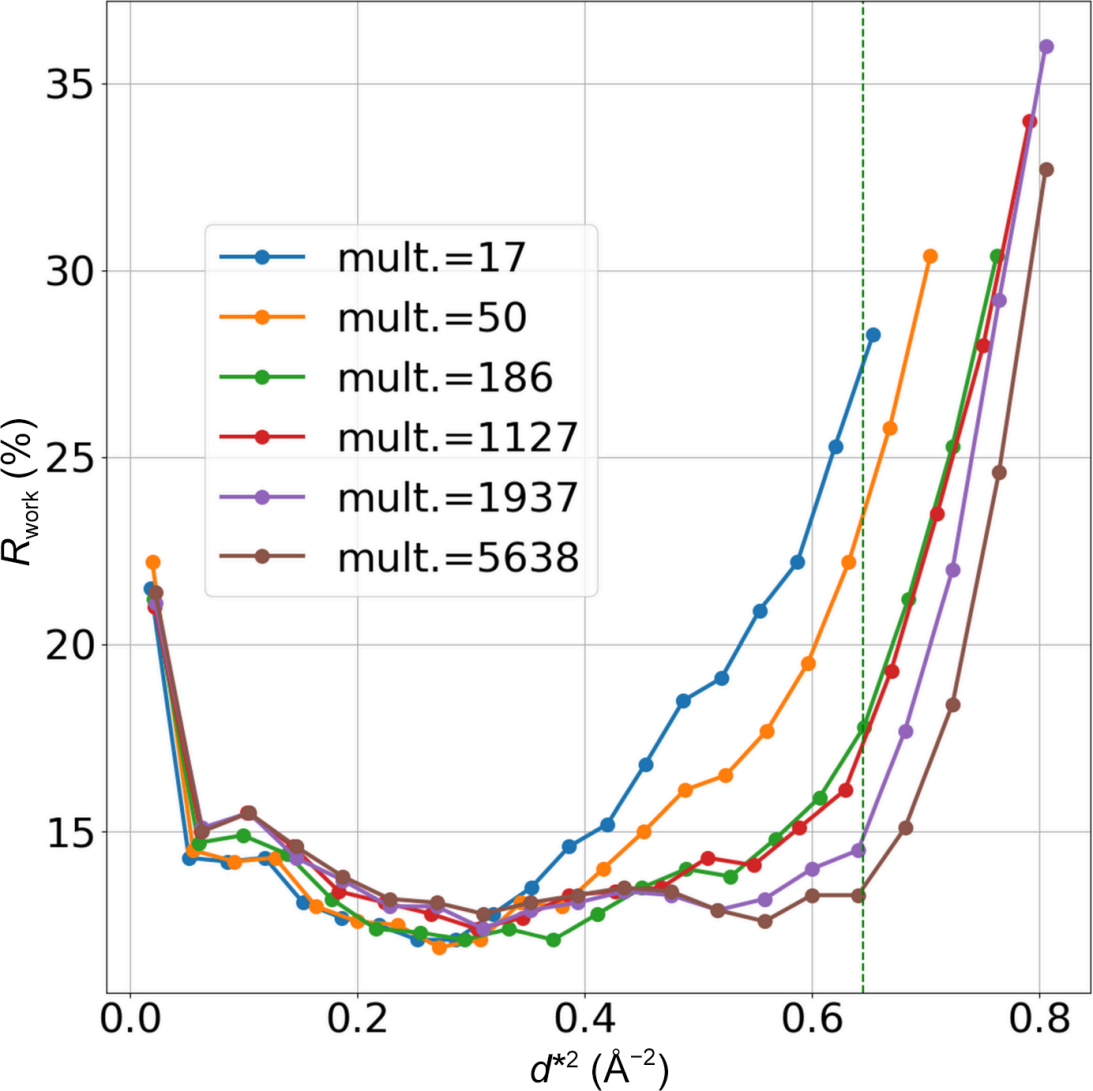
The *d*-dependency of *R*_work_ for representative subclusters of C11523. The ‘mlt’ values in the legend represent the multiplicity of the data set. The green vertical line shown indicates the resolution at the half corner of the detector, 1.25 Å.

Evaluating the mean atomic *B* factors calculated from refined PDB for 919 data sets, the mean, standard deviation, and median were 7.52, 1.86 and 7.20 Å^2^, respectively. These values were found to be significantly smaller compared with the mean, standard deviation and median of the mean temperature factors for 3556 data sets with resolutions of 1.05–1.20 Å registered in the PDB, which are 15.8, 4.3 and 15.5 Å^2^, respectively. This possibly suggests over-sharpening of the overall *B* factor (Masmaliyeva & Murshudov, 2019 ▸). Also, the mean overall *B* factor estimated by *CTRUNCATE* for 919 merged data sets in C11523 was 4.12 Å^2^, which is significantly lower than the typical values for 1.05–1.20 Å resolution in the PDB (mean, 11.9 Å^2^; median, 11.7 Å^2^). The Wilson plot curve for data set C1008 with a multiplicity of 17 appears to be normal, as shown in Fig. 4 ▸(*a*), yet the estimated Wilson *B* factor of 5.39 Å^2^ indicates over-sharpening. Examining the *d*-dependency of the 〈*I*/σ(*I*)〉 plot from the reflection files (Fig. 4 ▸*b*) revealed that even data sets with a flattened Wilson plot at higher resolution range seem to show proper signal accumulation. The *CTRUNCATE* logs showed fitting failures above ∼1.4 Å in all data sets for subclusters of C11523. Automatic data processing here depends on *CTRUNCATE*, and abnormally low atomic *B* factors indicated issues in this step. Refined models were re-refined with *REFMAC*, with 10 Å^2^*B*-blurred structure factors. After refinement, the mean, standard deviation and median *B* factors aligned with typical PDB values (16.6, 2.0 and 16.6 Å^2^, respectively). The *R*_free_ trends remained consistent, decreasing from 18.3% (mean) to 16.2% (mean) after *B*-blurred correction (Supplementary Fig. S3). Electron-density maps of representative subgroups showed no anomalies. Data and refinement statistics after the correction of both C11523 and C11524 are summarized in Supplementary Table S3. Based on the results of *B*-blurred refinement, the *d*-dependency of *R*_work_ was investigated using data from several subclusters of C11523 (Fig. 5 ▸). Gradually increasing the number of merged wedge data sets enhanced the resolution and reduced *R*_work_ at high resolutions. For the data set with a multiplicity of 5638, the Wilson plot exhibited unusual behavior at high resolutions (Fig. 4 ▸*a*). However, the *d*-dependency of the *R*-factor plot demonstrated that data-set merging effectively increased the structural information.

In *XSCALE*, relative *B* factors for wedge data sets are determined using a reference data set. *XSCALE*, run via *KAMO*, automatically selects the data set with the lowest *B* factor as the reference. If the reference data set is not properly chosen, *B*-factor inconsistencies can lead to over-sharpening or blurring. When merging large numbers of data sets, variations in crystal diffracting power and anisotropic X-ray exposure can affect multiplicity and σ estimation, particularly at high resolution. Many wedge sets in this study had resolution limits of around 1.5–1.7 Å based on a CC_1/2_ of ∼50%. Due to variations in diffracting power among crystals, some wedge data sets did not contribute to the higher resolution region, resulting in naturally reduced multiplicity in this range, which could finally lead to lower 〈*I*/σ(*I*)〉. Furthermore, in this experiment, incorrect camera-distance settings led to reduced completeness and multiplicity beyond the detector half corner (∼1.25 Å; details will be described at the end of this section). These combined factors are likely to contribute to inaccuracies in the estimation of the overall *B* factor. Therefore, as demonstrated in this study, it is essential to cross-check Wilson *B* factors and atomic *B*-factor values against PDB data to ensure consistency. If necessary, refinement should be performed using blurred or sharpened structure factors. The paired refinement approach used in this study proved to be one of the most effective methods for data assessment and is critical for identifying data sets that are suitable for accurate structural analysis.

**Figure 6:**
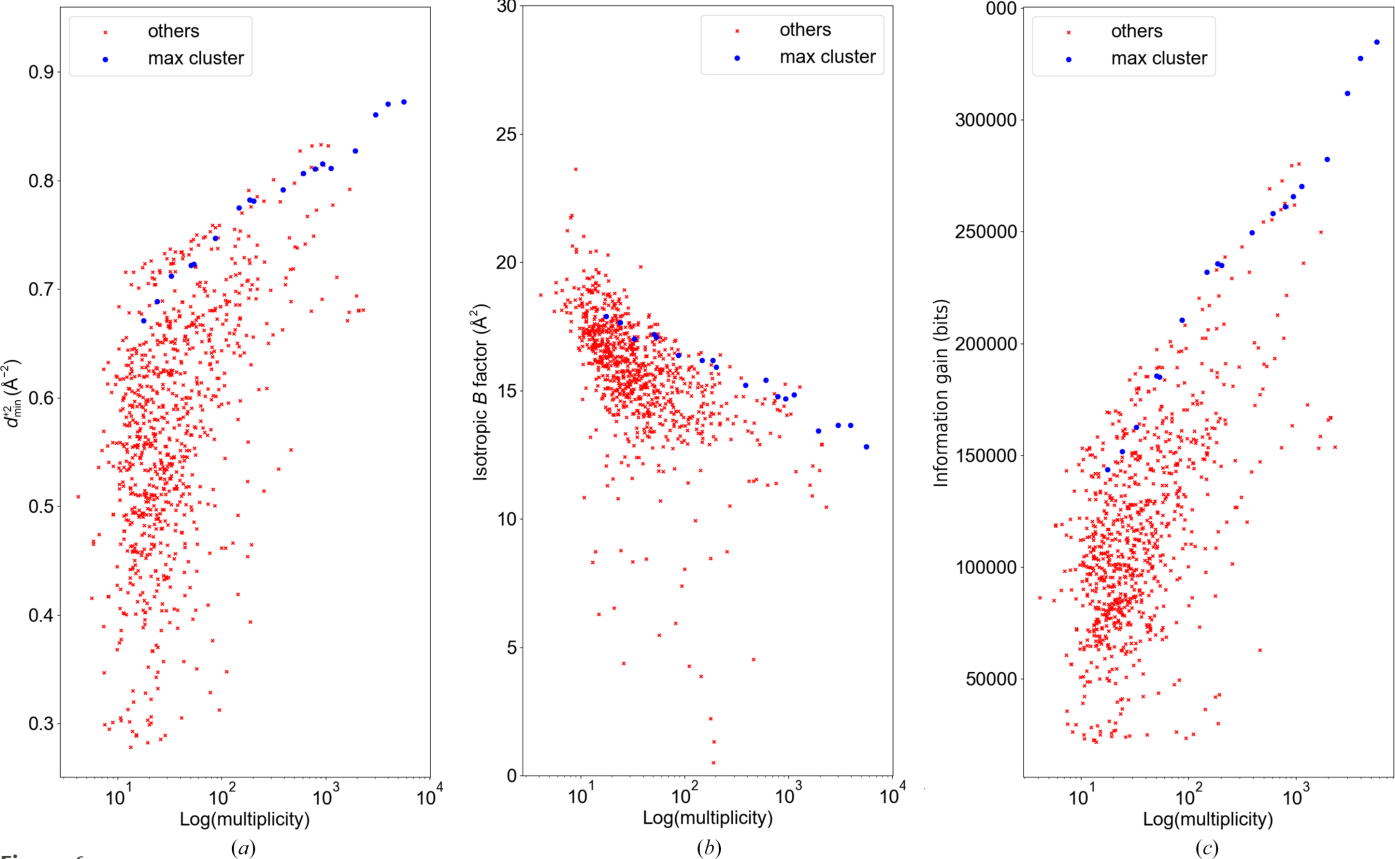
Statistics for the PhC (wedge = 10°) data set. The plots show (*a*) 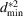, (*b*) isotropic *B* factor and (*c*) information gain from *Phaser*; the horizontal axis is the logarithm of multiplicity. All of the plots represent subgroups of C11523. The blue plots trace the subgroup that contains the largest number of data sets and represent the major component in C11523.

We plotted statistics of subclusters of C11523 in Fig. 6 ▸, which displays the relationship between multiplicity and 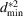, isotropic *B* factor and ‘information gain’ calculated by *Phaser*. All structural information improves with increasing multiplicity, as demonstrated experimentally, even at a multiplicity of 5638. The results roughly show a linear relationship between 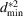, the isotropic *B* factor from refinement and the logarithm of multiplicity or the number of photons within this plane.

**Figure 7:**
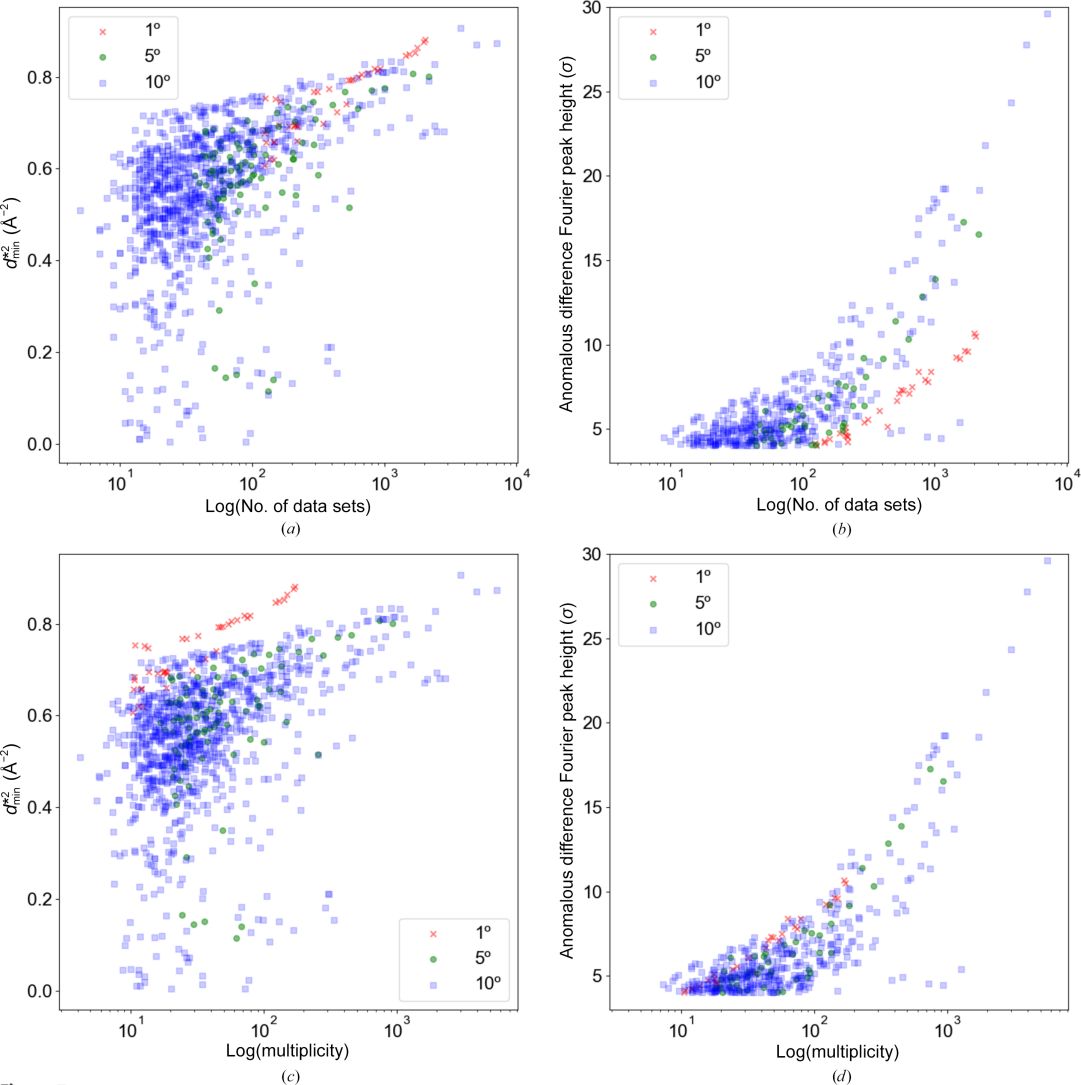
Comparison of data statistics with different PhC wedge sizes. (*a*, *b*) The logarithm of the number of merged data is taken on the abscissa. (*a*) The σ value of the anomalous signal observed at the S-atom position of Met124 and (*b*) 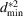 of each data set is plotted. (*c*, *d*) The abscissa shows the logarithm of multiplicity. (*c*) The σ value of the anomalous signal observed at the S-atom position of Met124 and (*d*) 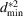 of each data set. The colors in the plots indicate wedge-size differences, with red, green and blue indicating wedge sizes of 1°, 5° and 10°, respectively.

PhC contains methionine in its sequence. The peak height of the anomalous difference Fourier map at the S atom of Met^124^ was examined using the method described in Section 2.3.2. When the σ value of the peak height in the anomalous difference Fourier map was plotted against the logarithm of multiplicity, the same trend was observed (Fig. 7 ▸). The σ value increases with higher multiplicity, continuing to increase up to a multiplicity of ∼5638. This experiment aimed to identify the limit of structural information improvement through merging, but no such limit was observed within this range.

In this experiment, the detector distance should have been set shorter, but due to beamline constraints it was set longer, resulting in the loss of some high-resolution data. Only about 20 out of 2876 merged data sets had an 〈*I*/σ(*I*)〉 exceeding 1.5 in the shell near *d*_min_, which is higher than the half-corner resolution of 1.25 Å. Considering the total number of data sets, the proportion of data sets affecting the overall trend is clearly small. Additionally, the plot in Fig. 6 ▸ does not indicate any unusual behavior in the high-resolution data, supporting the conclusion that structural information continues to increase within the current range of multiplicity. This suggests that the structural information, as quantified by the anomalous peak height, has not yet reached its limit. However, when aiming to merge a large number of data sets to accumulate signals, care must be taken to collect data with the camera distance as close as possible. Failing to do so may result in missing signals that could otherwise be accumulated.

#### 3.3.2. PhC comparison with different dose per rotation angle

To compare the structural information obtained from data sets with different wedge sizes but the same total dose, we analyzed 1°, 5° and 10° wedge data sets of PhC in this section.

Firstly, as observed in other experiments, both 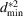 and anomalous signals improve with increasing multiplicity for data of any wedge size (Fig. 7 ▸). When comparing the number of wedge sets, both 1° and 10° wedges achieved similar *d*_min_ with the same amount of data (Fig. 7 ▸*a*). However, as shown in Fig. 7 ▸(*c*), when plotted against multiplicity, the 10° wedge data required ten times the multiplicity to achieve the same 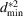 as the 1° wedge data, which is an expected outcome. Since the number of photons incident on each crystal is the same in this experiment, the number of merged data sets is linearly related to the total number of photons used for data collection. Therefore, it is natural to conclude that the resolution limit in this experiment is determined by the number of incident photons used to collect the final data, regardless of the wedge size.

Interestingly, this discussion about the number of photons does not apply to the amount of anomalous signal. Figs. 7 ▸(*b*) and 7 ▸(*d*) show the anomalous signal plotted against the logarithm of the number of wedge sets and the logarithm of reflection multiplicity, respectively. Fig. 7 ▸(*b*) indicates that the anomalous signal is significantly lower for 1° wedges compared with other wedge sizes. Furthermore, Fig. 7 ▸(*d*) shows that it is the multiplicity, not the number of incident photons, that clearly dictates the amount of anomalous signal. Therefore, to obtain the highest anomalous signal with the same number of crystals, it seems to be preferable to use a wedge size of 10° to increase the multiplicity. For reference, the average number of reflections measured in each wedge for the 1°, 5° and 10° data sets was 4758, 32 020 and 65 802, respectively. These averages were calculated from the number of full reflections recorded in the *DIALS* logs for representative data sets.

These considerations suggest that the choice of wedge size should be adjusted depending on the specific objectives. If the goal is to enhance resolution, it is effective to merge wedge sets collected with as many photons per angle as possible. On the other hand, if anomalous signal is essential, data collection with a larger wedge size is preferable.

As the wedge size decreases, there is less common information to compare, which can reduce the reliability of crystallographic isomorphism evaluation between wedge sets (crystals; Matsuura *et al.*, 2023 ▸). When there are concerns about crystal isomorphism, it is important to use larger wedge sizes in SWSX to improve clustering accuracy and robustly evaluate isomorphism. The PhC evaluated in this study belongs to a high-symmetry space group (*I*23), where even a 5° wedge can achieve about 44.5% completeness (median), which is an exceptional case. For crystals in lower symmetry space groups, it is expected that using larger wedge sizes will yield better results. Using larger wedge sizes not only facilitates the evaluation of isomorphism but also ensures high completeness with fewer crystals. The availability of homogeneously prepared crystals and their symmetry should also be considered in experimental settings.

The two conclusions mentioned above — that resolution is determined by the total number of photons used to collect the data sets and that higher multiplicity yields greater anomalous signals — may not always align with experimental observations. If both conclusions are always correct, it would imply that larger wedge sizes yield more anomalous signal with the same number of crystals and dose per crystal. For example, using microcrystals, collecting 360° rotation data per wedge would result in more anomalous signal, and despite the increased multiplicity the resolution limit would remain unchanged with the same number of incident photons. This seems unrealistic.

Therefore, this section concludes with the conditional finding that in SWSX with the same number of incident photons, similar resolution limits can be achieved after merging even if the wedge size varies by a factor of ten, and that higher multiplicity yields greater anomalous signals. More generally, it intuitively seems that if the dose per wedge is low diffraction signals may be lost, and important structural information might be compromised even if the multiplicity is increased. To understand this intuitive inconsistency more deeply, Section 3.4 investigates the relationship between weak diffraction intensity and multiplicity using dose slicing.

### 3.4. Dose-slicing results with thermolysin

**Figure 8:**
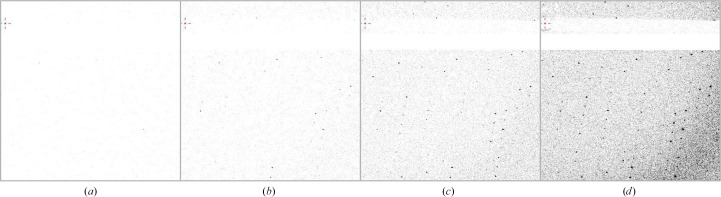
Diffraction images synthesized by summation using 50 kGy data sets. Diffraction images of (*a*) 50kGy, (*b*) v500kGy (ten frames summed), (*c*) v2.5MGy (50 frames summed) and (*d*) v5.0MGy (100 frames summed). Each frame contains 0.1° oscillation width. The beam center is located in the upper left corner of each image and is shown to approximately 2 Å resolution towards the lower right corner.

The images in Fig. 8 ▸ illustrate the simulated composite data sets described in Section 2.4.3 and Supplementary Fig. S1. The data collected at 50 kGy are at a very low dose, with signals that are not clearly visible even in the low-resolution regions (Fig. 8 ▸*a*). However, as the accumulation increases, diffraction spots in the high-resolution regions become visible (Figs. 8 ▸*b*, 8 ▸*c* and 8 ▸*d*). The v5.0MGy set reproduces almost the same resolution as the r5.0MGy data. This indicates that even with low photon incidence diffraction occurs on the crystal, and weak signals can be detected.

After visual inspection, the r5.0MGy and v5.0MGy data were subjected to standard data processing and analysis, and the main intensity statistics and structural information for each data set are shown in Table 3 ▸. While the resolution and the height of the anomalous difference peak are slightly lower for v5.0 MGy, almost equivalent structural information was reproduced. This indicates that even at 1% low-dose measurements, the EIGER X 9M can detect weak signals, especially in the higher resolution region. This data-processing result quantitatively aligns with the visual improvement of diffraction images through accumulation (Fig. 8 ▸). In the following, the v5.0MGy data will be used as a reference for comparison. This data set has a resolution of 1.50 Å, a refinement *R*_free_ factor of 22.9% and an anomalous difference peak of 45.3σ (Table 3 ▸). Data statistics and refinement details are summarized in Supplementary Table S4.

**Figure 9:**
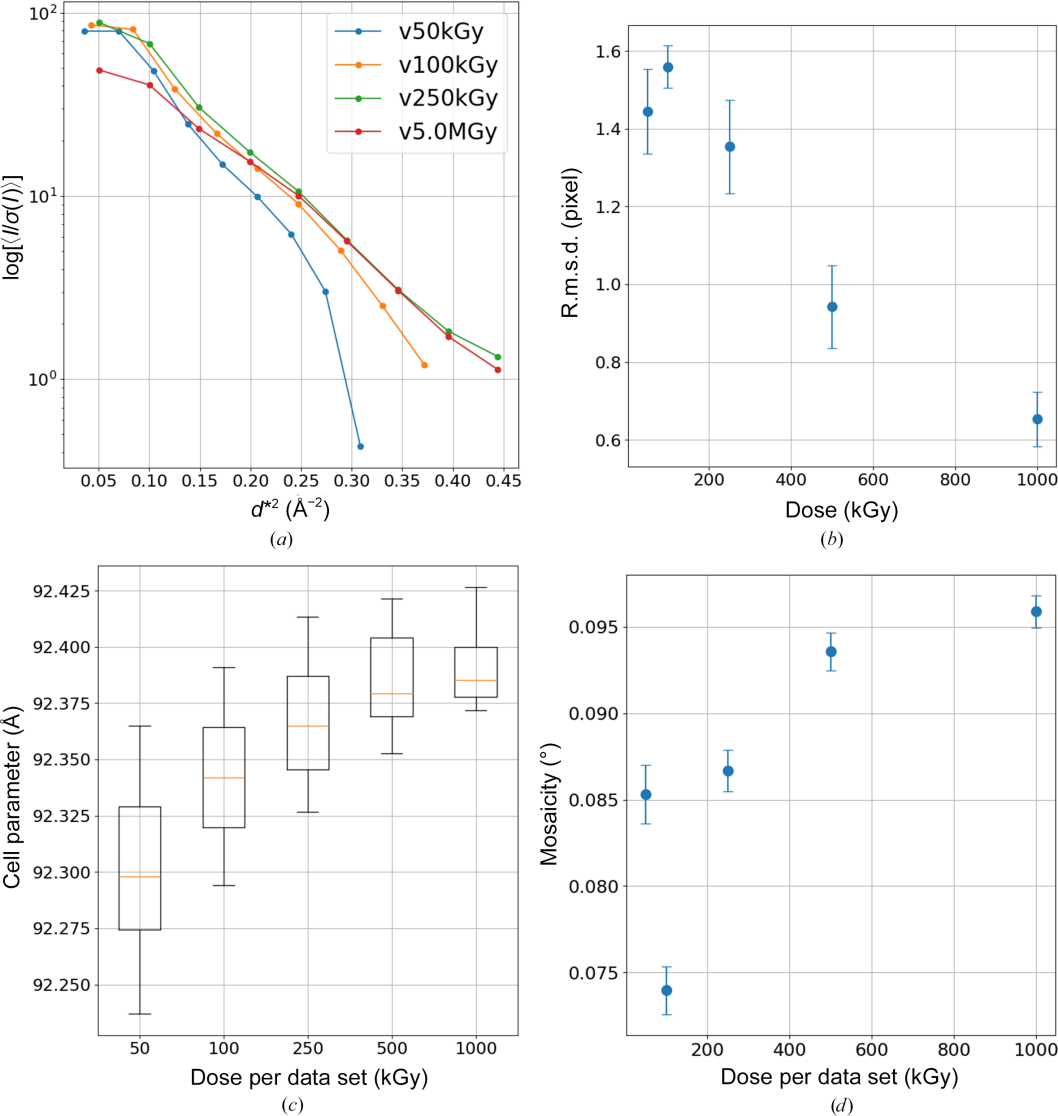
(*a*) Intensity statistics of dose-slicing data. (*b*) R.m.s.d. between the reflection model and observed data for each data set using *DIALS*. (*c*) Box plot of unit-cell parameters from *DIALS* processing for each data set. (*d*) Mosaicity of each data set estimated by *DIALS*. In (*b*), (*c*) and (*d*) the values are calculated from the sliced data sets of v50kGy, v100kGy, v250kGy, v500kGy and v1.0MGy using 100, 50, 20, 10 and 10 data sets, respectively, to obtain the mean and standard deviation. In (*b*) and (*d*), the standard deviation is displayed as error bars on both sides of the mean value.

The resolution limit was 1.80 Å when 100 data sets of 50 kGy were merged, and the peak of the anomalous difference Fourier map at the zinc position deteriorated to ∼39.5σ. The *R*_free_ factor for the 50 kGy data was 36.8%, which was not appropriate for the obtained resolution. Fig. 9 ▸(*a*) shows the 〈*I*/σ(*I*)〉 plot of the 50kGy, v100kGy, v250kGy and v5.0MGy data sets. It suggests that the 50 kGy and 100 kGy data lose signal in the high-resolution region. When more frames are summed, the 〈*I*/σ(*I*)〉 approaches that of the v5.0MGy data, indicating that the diffraction intensity required for recovery exists in the images. The resolution of the v100kGy data is not fully recovered at 1.64 Å, but the structural refinement and anomalous difference peak heights are almost equivalent to the high-dose data. In the v250kGy data, with the dose reduced to about 1/20 of the original, the signal was nearly fully recovered by merging 20 sets, resulting in structural information equivalent to the v5.0MGy data.

The series with a lower dose than 250 kGy did not adequately acquire the intensity to achieve comparable statistical values. We plotted the root-mean-square deviation (r.m.s.d.) of model and observed reflections during integration against dose per frame (Fig. 9 ▸*b*). As a result, the r.m.s.d. was larger for low-dose data, suggesting potential integration errors. We also compared the values of the unit-cell constants *a* and *b* in space group *P*6_1_22 among data sets using a box plot (Fig. 9 ▸*c*). Although very slight, the trend indicates that lower doses estimate shorter unit-cell constants with greater variance, suggesting lower precision in parameter estimation for low-intensity data. Mosaicity also shows lower estimates for low-dose data compared with high-dose data (Fig. 9 ▸*d*), potentially resulting in incorrect models for profile fitting and reduced the data-processing quality.

These results indicate that even weak diffraction intensities, when accurately integrated, can increase the structural information through the accumulation of signals by merging data sets. However, as the number of incident photons decreases, data processing becomes increasingly difficult. Particularly for low-dose data, pixel values around the diffraction spots approach zero background, making it challenging to accurately determine parameters such as unit-cell constants and mosaicity. Additionally, low-dose data tend to lose the tails of diffraction intensities, complicating profile modeling and making it more difficult to accurately integrate the diffraction spots.

The conclusion of the previous section was that within the range of wedge sizes from 1° to 10° the resolution limit is determined by the number of incident photons, and higher multiplicity yields more anomalous signals. However, the results of this section show that reducing the dose per data set to 1/100 and increasing the multiplicity 100 times leads to a loss of structural information. Specifically, in this experiment, when the number of incident photons was reduced to less than 1/20, even increasing the multiplicity until the total number of photons matched the original data set did not fully recover the original data, indicating a loss of signal.

Based on the above validation results, it is easy to imagine that when merging a large number of data sets, the impact of signal loss in individual data processing becomes more significant on the overall data accuracy compared with merging a small number of data sets.

## 4. Conclusion and remarks

The results presented in this paper are as follows. Firstly, the strategy of ‘utilizing multiple crystals’ is shown to be extremely significant for low-resolution data of membrane proteins, phase determination of membrane proteins and high-resolution data collection from microcrystals. Additionally, we evaluated whether there is an upper limit to the structural information that can be obtained by signal accumulation, and quantified and assessed improvements in structural information, such as resolution limit and anomalous signals. As a result, structural information was found to increase with multiplicity for all samples, continuing to increase even when the multiplicity reached approximately 5638. In our experiments, no plateau was observed in the increase of structural information within this range, suggesting that adding more data could further increase the structural information. Based on the results of this study, it is recommended that future experiments conduct measurements with the detector positioned at a distance significantly shorter than that required to cover the apparent diffracting power of the crystal. This approach would allow a more thorough evaluation of the observed trend in which structural information increases with multiplicity without reaching a saturation point.

From these results, it can be inferred that structural information has a linear correlation with the logarithm of multiplicity, allowing the design of optimized experimental workflows. This applies to phase determination using the SWSX method as well, where redundant data should be collected to improve phase quality, especially when a certain level of isomorphism is ensured. This approach is also considered to be effective for precise structural analysis, such as capturing smaller structural changes.

Furthermore, the results from the PhC 1–10° SWSX experiments showed that even if the number of incident photons per oscillation angle differs, the obtained resolution remains almost identical if the total number of incident photons is identical. To simply improve the resolution in native data, increasing the total number of photons is likely to yield good results. Moreover, anomalous signals are governed not by the total number of incident photons but by the multiplicity. To enhance the detection of anomalous signals, priority should be given to increasing the multiplicity in measurements. However, careful consideration must also be given to the number of photons per oscillation angle, as this can impact the accuracy of data processing. For instance, when increasing the oscillation range of measurement to enhance multiplicity, it is recommended to select conditions that ensure the number of photons per oscillation angle does not fall below 1/20 of the initial conditions. This approach will help to maintain the accuracy of data processing.

Handling crystal isomorphism in large data sets is challenging and requires care. All of the samples in this study showed potential polymorphism. Hierarchical clustering is crucial for refining structures and evaluating electron-density maps before further merging. Metrics such as the change in *R* value at high resolution, as in paired refinement, are effective. Accurate grouping of isomorphic data sets is essential for isomorphous merging, particularly when dealing with small wedge sizes or low-symmetry crystals that require special attention. Limited shared crystallographic information in such cases complicates isomorphism quantification. Larger wedge sizes are preferable if isomorphism is a concern. In large-scale data merging, it is also important to evaluate the data from multiple perspectives, such as the Wilson plot shape and the average *B* factor after refinement.

Accurately integrating weak diffraction signals using data-processing programs is crucial to completely extract information from crystals. Particularly when integrating a large amount of data, summing weak diffraction signals from each data set can yield useful structural information, significantly affecting the results of structural analysis. Techniques for integrating very weak diffraction intensities will continue to become increasingly important in the future, especially in room-temperature measurements or other experiments that are sensitive to radiation damage. Developing methods for low-dose options will be essential for broadening experimental capabilities and ensuring the accurate integration of weak signals.

Finally, based on the results of this study, we propose the optimal rotation range for SWSX using an X-ray beam size of 5–10 µm. The collection of each wedge of data depends on the positional reproducibility of the beamline equipment. To prevent misalignment between the beam and the crystal due to rotation, it is advisable to limit the oscillation range to 10° or less. From the results of this study, if the dose for data collection is kept constant and the wedge size is set to 10° or less, the resolution should be the same whether using 1° or 5° wedge sizes, provided that the number of photons is equivalent. However, to broaden the applicability, a larger wedge size is preferable for robust evaluation of isomorphism and stable acquisition of high-resolution data and anomalous signals, even for low-symmetry crystals. Therefore, as an initial choice, we recommend trying SWSX with a wedge size of 10°.

## 5. Availability

The analysis presented here can be performed anywhere by installing the program *KAMO*, which is available from GitHub (https://github.com/keitaroyam/yamtbx). The raw diffraction data used in this study are available on Zenodo and XRD-Arc. The 100 data sets of 50 kGy repeatedly collected using thermolysin crystals can be accessed at https://doi.org/10.5281/zenodo.13234452. Data sets for AT_2_R, CNNM/CorC and PhC are available at https://doi.org/10.51093/xrd-00276, https://doi.org/10.51093/xrd-00275 and https://doi.org/10.51093/xrd-00277, respectively.

## Supplementary Material

Supplementary Figures and Tables. DOI: 10.1107/S2059798324011987/wa5149sup1.pdf

## Figures and Tables

**Table 1:** Summary of intensity and refinement statistics for each cluster of AT_2_R

Cluster ID	No. of data sets	*d*_min_ (Å)	Multiplicity	*R*_free_ (%)	*R*_work_ (%)
3203	250	3.86	46.3	29.6	24.1
3219	216	3.77	39.8	31.1	25.1
3241	31	10.0	5.1	45.1	35.0
3244	166	3.88	30.6	31.0	24.1
3245	146	4.05	26.7	30.6	24.9
3251	329	3.56	60.8	30.4	24.8
3252	1545	3.26	283.5	30.6	24.8
3254	1873	3.24	343.2	31.2	24.8
3255	2038	3.21	373.4	30.5	24.7
3256	2194	3.21	401.8	31.6	25.2
3257	2421	3.20	443.0	31.2	25.2
3258	2665	3.19	487.0	31.5	25.2

**Table 2:** Summary of intensity and refinement statistics for each cluster of CNNM/CorC

Cluster ID	No. of data sets	*d*_min_ (Å)	CC_map_	Multiplicity	*R*_free_ (%)	*R*_work_ (%)
1278	182	2.66	0.256	31.5	27.2	24.6
1312	187	2.74	0.190	31.8	27.5	24.7
1316	75	3.49	0.016	12.7	55.1	44.3
1318	508	2.45	0.614	85.0	25.9	24.4
1319	179	2.69	0.181	30.5	27.8	25.2
1320	700	2.35	0.689	121.3	31.5	29.9
1321	863	2.33	0.728	149.0	33.5	30.0
1322	254	2.72	0.137	43.0	27.5	24.8
1323	1122	2.33	0.726	194.0	30.3	28.2

**Table 3:** Summary of intensity statistics and structural information for data synthesized from 50 kGy low-dose data with r5.0MGy data

Data name	No. of data sets	Multiplicity	*d*_min_ (Å)	Overall *R*_p.i.m._ (%)	*R*_free_ (%)	Anomalous difference Fourier peak height (σ)
r5.0MGy	1	39.0	1.50	5.6	22.6	47.8
v5.0MGy	1	38.7	1.50	4.8	22.9	45.3
50kGy	100	3967.3	1.80	2.6	36.8	39.5
v100kGy	50	1961.4	1.64	3.1	20.8	45.1
v250kGy	20	784.3	1.50	3.5	22.5	45.3
v500kGy	10	391.1	1.55	4.4	23.8	44.3
v1.0MGy	5	195.6	1.50	4.4	22.7	41.3

## References

[bb1] Abe, S., Pham, T. T., Negishi, H., Yamashita, K., Hirata, K. & Ueno, T. (2021). *Angew. Chem. Int. Ed.* **60**, 12341–12345. 10.1002/anie.202102039 33759310

[bb2] Abe, S., Tanaka, J., Kojima, M., Kanamaru, S., Hirata, K., Yamashita, K., Kobayashi, A. & Ueno, T. (2022). *Sci. Rep.* **12**, 16031. 10.1038/s41598-022-19681-9 PMC9530169 36192567

[bb3] Agirre, J., Atanasova, M., Bagdonas, H., Ballard, C. B., Baslé, A., Beilsten-Edmands, J., Borges, R. J., Brown, D. G., Burgos-Mármol, J. J., Berrisford, J. M., Bond, P. S., Caballero, I., Catapano, L., Chojnowski, G., Cook, A. G., Cowtan, K. D., Croll, T. I., Debreczeni, J. É., Devenish, N. E., Dodson, E. J., Drevon, T. R., Emsley, P., Evans, G., Evans, P. R., Fando, M., Foadi, J., Fuentes-Montero, L., Garman, E. F., Gerstel, M., Gildea, R. J., Hatti, K., Hekkelman, M. L., Heuser, P., Hoh, S. W., Hough, M. A., Jenkins, H. T., Jiménez, E., Joosten, R. P., Keegan, R. M., Keep, N., Krissinel, E. B., Kolenko, P., Kovalevskiy, O., Lamzin, V. S., Lawson, D. M., Lebedev, A. A., Leslie, A. G. W., Lohkamp, B., Long, F., Malý, M., McCoy, A. J., McNicholas, S. J., Medina, A., Millán, C., Murray, J. W., Murshudov, G. N., Nicholls, R. A., Noble, M. E. M., Oeffner, R., Pannu, N. S., Parkhurst, J. M., Pearce, N., Pereira, J., Perrakis, A., Powell, H. R., Read, R. J., Rigden, D. J., Rochira, W., Sammito, M., Sánchez Rodríguez, F., Sheldrick, G. M., Shelley, K. L., Simkovic, F., Simpkin, A. J., Skubak, P., Sobolev, E., Steiner, R. A., Stevenson, K., Tews, I., Thomas, J. M. H., Thorn, A., Valls, J. T., Uski, V., Usón, I., Vagin, A., Velankar, S., Vollmar, M., Walden, H., Waterman, D., Wilson, K. S., Winn, M. D., Winter, G., Wojdyr, M. & Yamashita, K. (2023). *Acta Cryst.* D**79**, 449–461.

[bb4] Asada, H., Horita, S., Hirata, K., Shiroishi, M., Shiimura, Y., Iwanari, H., Hamakubo, T., Shimamura, T., Nomura, N., Kusano-Arai, O., Uemura, T., Suno, C., Kobayashi, T. & Iwata, S. (2018). *Nat. Struct. Mol. Biol.* **25**, 570–576. 10.1038/s41594-018-0079-8 29967536

[bb5] Baba, S., Matsuura, H., Kawamura, T., Sakai, N., Nakamura, Y., Kawano, Y., Mizuno, N., Kumasaka, T., Yamamoto, M. & Hirata, K. (2021). *J. Synchrotron Rad.* **28**, 1284–1295. 10.1107/S1600577521008067 PMC8415328 34475278

[bb6] Basu, S., Kaminski, J. W., Panepucci, E., Huang, C.-Y., Warshamanage, R., Wang, M. & Wojdyla, J. A. (2019). *J. Synchrotron Rad.* **26**, 244–252. 10.1107/S1600577518016570 PMC6337882 30655492

[bb7] Basu, S., Olieric, V., Leonarski, F., Matsugaki, N., Kawano, Y., Takashi, T., Huang, C.-Y., Yamada, Y., Vera, L., Olieric, N., Basquin, J., Wojdyla, J. A., Bunk, O., Diederichs, K., Yamamoto, M. & Wang, M. (2019). *IUCrJ*, **6**, 373–386. 10.1107/S2052252519002756 PMC6503925 31098019

[bb8] Beyerlein, K. R., Dierksmeyer, D., Mariani, V., Kuhn, M., Sarrou, I., Ottaviano, A., Awel, S., Knoska, J., Fuglerud, S., Jönsson, O., Stern, S., Wiedorn, M. O., Yefanov, O., Adriano, L., Bean, R., Burkhardt, A., Fischer, P., Heymann, M., Horke, D. A., Jungnickel, K. E. J., Kovaleva, E., Lorbeer, O., Metz, M., Meyer, J., Morgan, A., Pande, K., Panneerselvam, S., Seuring, C., Tolstikova, A., Lieske, J., Aplin, S., Roessle, M., White, T. A., Chapman, H. N., Meents, A. & Oberthuer, D. (2017). *IUCrJ*, **4**, 769–777. 10.1107/S2052252517013124 PMC5668862 29123679

[bb9] Caffrey, M. (2003). *J. Struct. Biol.* **142**, 108–132. 10.1016/s1047-8477(03)00043-1 12718924

[bb10] Coulibaly, F., Chiu, E., Ikeda, K., Gutmann, S., Haebel, P. W., Schulze-Briese, C., Mori, H. & Metcalf, P. (2007). *Nature*, **446**, 97–101. 10.1038/nature05628 17330045

[bb11] Evans, G., Axford, D. & Owen, R. L. (2011). *Acta Cryst.* D**67**, 261–270. 10.1107/S0907444911007608 PMC3069741 21460444

[bb12] Foadi, J., Aller, P., Alguel, Y., Cameron, A., Axford, D., Owen, R. L., Armour, W., Waterman, D. G., Iwata, S. & Evans, G. (2013). *Acta Cryst.* D**69**, 1617–1632. 10.1107/S0907444913012274 PMC3727331 23897484

[bb13] Gavira, J. A., Rodriguez-Ruiz, I., Martinez-Rodriguez, S., Basu, S., Teychené, S., McCarthy, A. A. & Mueller-Dieckman, C. (2020). *Acta Cryst.* D**76**, 751–758. 10.1107/S2059798320008475 32744257

[bb14] Gildea, R. J., Beilsten-Edmands, J., Axford, D., Horrell, S., Aller, P., Sandy, J., Sanchez-Weatherby, J., Owen, C. D., Lukacik, P., Strain-Damerell, C., Owen, R. L., Walsh, M. A. & Winter, G. (2022). *Acta Cryst.* D**78**, 752–769. 10.1107/S2059798322004399 PMC9159281 35647922

[bb15] Giordano, R., Leal, R. M. F., Bourenkov, G. P., McSweeney, S. & Popov, A. N. (2012). *Acta Cryst.* D**68**, 649–658. 10.1107/S0907444912006841 22683787

[bb16] Hasegawa, K., Yamashita, K., Murai, T., Nuemket, N., Hirata, K., Ueno, G., Ago, H., Nakatsu, T., Kumasaka, T. & Yamamoto, M. (2017). *J. Synchrotron Rad.* **24**, 29–41. 10.1107/S1600577516016362 PMC5182019 28009544

[bb17] Henrich, B., Bergamaschi, A., Broennimann, C., Dinapoli, R., Eikenberry, E. F., Johnson, I., Kobas, M., Kraft, P., Mozzanica, A. & Schmitt, B. (2009). *Nucl. Instrum. Methods Phys. Res. A*, **607**, 247–249.

[bb18] Hirata, K., Kawano, Y., Ueno, G., Hashimoto, K., Murakami, H., Hasegawa, K., Hikima, T., Kumasaka, T. & Yamamoto, M. (2013). *J. Phys. Conf. Ser.* **425**, 012002.

[bb19] Hirata, K., Yamashita, K., Ueno, G., Kawano, Y., Hasegawa, K., Kumasaka, T. & Yamamoto, M. (2019). *Acta Cryst.* D**75**, 138–150. 10.1107/S2059798318017795 PMC6400253 30821703

[bb20] Huang, Y., Jin, F., Funato, Y., Xu, Z., Zhu, W., Wang, J., Sun, M., Zhao, Y., Yu, Y., Miki, H. & Hattori, M. (2021). *Sci. Adv.* **7**, eabe6140. 10.1126/sciadv.abe6140 PMC7875539 33568487

[bb21] Kabsch, W. (2010). *Acta Cryst.* D**66**, 125–132. 10.1107/S0907444909047337 PMC2815665 20124692

[bb22] Kabsch, W. (2014). *Acta Cryst.* D**70**, 2204–2216. 10.1107/S1399004714013534 PMC4118830 25084339

[bb23] Kato, H. E., Zhang, F., Yizhar, O., Ramakrishnan, C., Nishizawa, T., Hirata, K., Ito, J., Aita, Y., Tsukazaki, T., Hayashi, S., Hegemann, P., Maturana, A. D., Ishitani, R., Deisseroth, K. & Nureki, O. (2012). *Nature*, **482**, 369–374. 10.1038/nature10870 PMC4160518 22266941

[bb24] Kumazaki, K., Chiba, S., Takemoto, M., Furukawa, A., Nishiyama, K.-I., Sugano, Y., Mori, T., Dohmae, N., Hirata, K., Nakada-Nakura, Y., Maturana, A. D., Tanaka, Y., Mori, H., Sugita, Y., Arisaka, F., Ito, K., Ishitani, R., Tsukazaki, T. & Nureki, O. (2014). *Nature*, **509**, 516–520. 10.1038/nature13167 24739968

[bb25] Liu, Q., Liu, Q. & Hendrickson, W. A. (2013). *Acta Cryst.* D**69**, 1314–1332. 10.1107/S0907444913001479 PMC3689535 23793158

[bb26] Liu, Z.-J., Chen, L., Wu, D., Ding, W., Zhang, H., Zhou, W., Fu, Z.-Q. & Wang, B.-C. (2011). *Acta Cryst.* A**67**, 544–549. 10.1107/S0108767311037469 PMC3211246 22011470

[bb27] Marin, E., Luginina, A., Gusach, A., Kovalev, K., Bukhdruker, S., Khorn, P., Polovinkin, V., Lyapina, E., Rogachev, A., Gordeliy, V., Mishin, A., Cherezov, V. & Borshchevskiy, V. (2020). *Sci. Data*, **7**, 388. 10.1038/s41597-020-00729-2 PMC7661540 33184270

[bb28] Martin-Garcia, J. M., Conrad, C. E., Nelson, G., Stander, N., Zatsepin, N. A., Zook, J., Zhu, L., Geiger, J., Chun, E., Kissick, D., Hilgart, M. C., Ogata, C., Ishchenko, A., Nagaratnam, N., Roy-Chowdhury, S., Coe, J., Subramanian, G., Schaffer, A., James, D., Ketwala, G., Venugopalan, N., Xu, S., Corcoran, S., Ferguson, D., Weierstall, U., Spence, J. C. H., Cherezov, V., Fromme, P., Fischetti, R. F. & Liu, W. (2017). *IUCrJ*, **4**, 439–454. 10.1107/S205225251700570X PMC5571807 28875031

[bb29] Masmaliyeva, R. C. & Murshudov, G. N. (2019). *Acta Cryst.* D**75**, 505–518. 10.1107/S2059798319004807 PMC6503761 31063153

[bb30] Matsuura, H., Sakai, N., Toma-Fukai, S., Muraki, N., Hayama, K., Kamikubo, H., Aono, S., Kawano, Y., Yamamoto, M. & Hirata, K. (2023). *Acta Cryst.* D**79**, 909–924. 10.1107/S2059798323007039 PMC10565733 37747037

[bb31] Mueller, M., Wang, M. & Schulze-Briese, C. (2012). *Acta Cryst.* D**68**, 42–56. 10.1107/S0907444911049833 PMC3245722 22194332

[bb32] Murshudov, G. N., Skubák, P., Lebedev, A. A., Pannu, N. S., Steiner, R. A., Nicholls, R. A., Winn, M. D., Long, F. & Vagin, A. A. (2011). *Acta Cryst.* D**67**, 355–367. 10.1107/S0907444911001314 PMC3069751 21460454

[bb33] Nanao, M., Basu, S., Zander, U., Giraud, T., Surr, J., Guijarro, M., Lentini, M., Felisaz, F., Sinoir, J., Morawe, C., Vivo, A., Beteva, A., Oscarsson, M., Caserotto, H., Dobias, F., Flot, D., Nurizzo, D., Gigmes, J., Foos, N., Siebrecht, R., Roth, T., Theveneau, P., Svensson, O., Papp, G., Lavault, B., Cipriani, F., Barrett, R., Clavel, C. & Leonard, G. (2022). *J. Synchrotron Rad.* **29**, 581–590.

[bb34] Nguyen, T., Phan, K. L., Kozakov, D., Gabelli, S. B., Kreitler, D. F., Andrews, L. C., Jakoncic, J., Sweet, R. M., Soares, A. S. & Bernstein, H. J. (2022). *Acta Cryst.* D**78**, 268–277. 10.1107/S2059798321013425 PMC8900820 35234141

[bb35] Nishizawa, T., Kita, S., Maturana, A. D., Furuya, N., Hirata, K., Kasuya, G., Ogasawara, S., Dohmae, N., Iwamoto, T., Ishitani, R. & Nureki, O. (2013). *Science*, **341**, 168–172. 10.1126/science.1239002 23704374

[bb36] Owen, R. L., Axford, D., Sherrell, D. A., Kuo, A., Ernst, O. P., Schulz, E. C., Miller, R. J. D. & Mueller-Werkmeister, H. M. (2017). *Acta Cryst.* D**73**, 373–378. 10.1107/S2059798317002996 PMC5379936 28375148

[bb37] Paithankar, K. S. & Garman, E. F. (2010). *Acta Cryst.* D**66**, 381–388. 10.1107/S0907444910006724 PMC2852302 20382991

[bb38] Read, R. J., Oeffner, R. D. & McCoy, A. J. (2020). *Acta Cryst.* D**76**, 238–247. 10.1107/S2059798320001588 PMC7057217 32133988

[bb39] Rose, J. P., Wang, B.-C. & Weiss, M. S. (2015). *IUCrJ*, **2**, 431–440. 10.1107/S2052252515008337 PMC4491315 26175902

[bb40] Santoni, G., Zander, U., Mueller-Dieckmann, C., Leonard, G. & Popov, A. (2017). *J. Appl. Cryst.* **50**, 1844–1851. 10.1107/S1600576717015229 PMC5713145 29217993

[bb41] Schneider, D. K., Soares, A. S., Lazo, E. O., Kreitler, D. F., Qian, K., Fuchs, M. R., Bhogadi, D. K., Antonelli, S., Myers, S. S., Martins, B. S., Skinner, J. M., Aishima, J., Bernstein, H. J., Langdon, T., Lara, J., Petkus, R., Cowan, M., Flaks, L., Smith, T., Shea-McCarthy, G., Idir, M., Huang, L., Chubar, O., Sweet, R. M., Berman, L. E., McSweeney, S. & Jakoncic, J. (2022). *J. Synchrotron Rad.* **29**, 1480–1494. 10.1107/S1600577522009377 PMC9641562 36345756

[bb42] Sheldrick, G. M. (2015). *Acta Cryst.* C**71**, 3–8.

[bb43] Smith, J. L., Fischetti, R. F. & Yamamoto, M. (2012). *Curr. Opin. Struct. Biol.* **22**, 602–612. 10.1016/j.sbi.2012.09.001 PMC3478446 23021872

[bb44] Soares, A. S., Yamada, Y., Jakoncic, J., McSweeney, S., Sweet, R. M., Skinner, J., Foadi, J., Fuchs, M. R., Schneider, D. K., Shi, W., Andi, B., Andrews, L. C. & Bernstein, H. J. (2022). *Acta Cryst.* F**78**, 281–288. 10.1107/S2053230X22006422 PMC9254899 35787556

[bb45] Stellato, F., Oberthür, D., Liang, M., Bean, R., Gati, C., Yefanov, O., Barty, A., Burkhardt, A., Fischer, P., Galli, L., Kirian, R. A., Meyer, J., Panneerselvam, S., Yoon, C. H., Chervinskii, F., Speller, E., White, T. A., Betzel, C., Meents, A. & Chapman, H. N. (2014). *IUCrJ*, **1**, 204–212. 10.1107/S2052252514010070 PMC4107920 25075341

[bb55] Thorn, A. & Sheldrick, G. M. (2011). *J. Appl. Cryst.* **44**, 1285–1287. 10.1107/S0021889811041768 PMC3246834 22477786

[bb46] Ursby, T., Åhnberg, K., Appio, R., Aurelius, O., Barczyk, A., Bartalesi, A., Bjelčić, M., Bolmsten, F., Cerenius, Y., Doak, R. B., Eguiraun, M., Eriksson, T., Friel, R. J., Gorgisyan, I., Gross, A., Haghighat, V., Hennies, F., Jagudin, E., Norsk Jensen, B., Jeppsson, T., Kloos, M., Lidon-Simon, J., de Lima, G. M. A., Lizatovic, R., Lundin, M., Milan-Otero, A., Milas, M., Nan, J., Nardella, A., Rosborg, A., Shilova, A., Shoeman, R. L., Siewert, F., Sondhauss, P., Talibov, V. O., Tarawneh, H., Thånell, J., Thunnissen, M., Unge, J., Ward, C., Gonzalez, A. & Mueller, U. (2020). *J. Synchrotron Rad.* **27**, 1415–1429. 10.1107/S1600577520008723 PMC7467343 32876619

[bb47] Winter, G., Gildea, R. J., Paterson, N., Beale, J., Gerstel, M., Axford, D., Vollmar, M., McAuley, K. E., Owen, R. L., Flaig, R., Ashton, A. W. & Hall, D. R. (2019). *Acta Cryst.* D**75**, 242–261. 10.1107/S2059798319003528 PMC6450062 30950396

[bb48] Winter, G., Waterman, D. G., Parkhurst, J. M., Brewster, A. S., Gildea, R. J., Gerstel, M., Fuentes-Montero, L., Vollmar, M., Michels-Clark, T., Young, I. D., Sauter, N. K. & Evans, G. (2018). *Acta Cryst.* D**74**, 85–97. 10.1107/S2059798317017235 PMC5947772 29533234

[bb49] Yamamoto, M., Hirata, K., Yamashita, K., Hasegawa, K., Ueno, G., Ago, H. & Kumasaka, T. (2017). *IUCrJ*, **4**, 529–539. 10.1107/S2052252517008193 PMC5619846 28989710

[bb50] Yamashita, K., Hirata, K. & Yamamoto, M. (2018). *Acta Cryst.* D**74**, 441–449. 10.1107/S2059798318004576 PMC5930351 29717715

[bb51] Zander, U., Bourenkov, G., Popov, A. N., de Sanctis, D., Svensson, O., McCarthy, A. A., Round, E., Gordeliy, V., Mueller-Dieckmann, C. & Leonard, G. A. (2015). *Acta Cryst.* D**71**, 2328–2343. 10.1107/S1399004715017927 PMC4631482 26527148

[bb52] Zwart, P. H., Afonine, P. V., Grosse-Kunstleve, R. W., Hung, L.-W., Ioerger, T. R., McCoy, A. J., McKee, E., Moriarty, N. W., Read, R. J., Sacchettini, J. C., Sauter, N. K., Storoni, L. C., Terwilliger, T. C. & Adams, P. D. (2008). *Methods Mol. Biol.* **426**, 419–435. 10.1007/978-1-60327-058-8_28 18542881

