# Supplementary material for: Useful experimental aspects of small-wedge synchrotron crystallography for accurate structure analysis of protein molecules
Source: Acta Crystallogr D Struct Biol. 2025 Jan 1;81(Pt 1):22–37. doi: 10.1107/S2059798324011987 (PMC11740584; doi:10.1107/S2059798324011987)
Supplement: Supplementary file 1 [file d-81-00022-sup1.pdf]

Volume 81 (2025)

**Supporting information for article:**

# Useful experimental aspects of small-wedge synchrotron crystallography for accurate structure analysis of protein molecules

Kunio Hirata

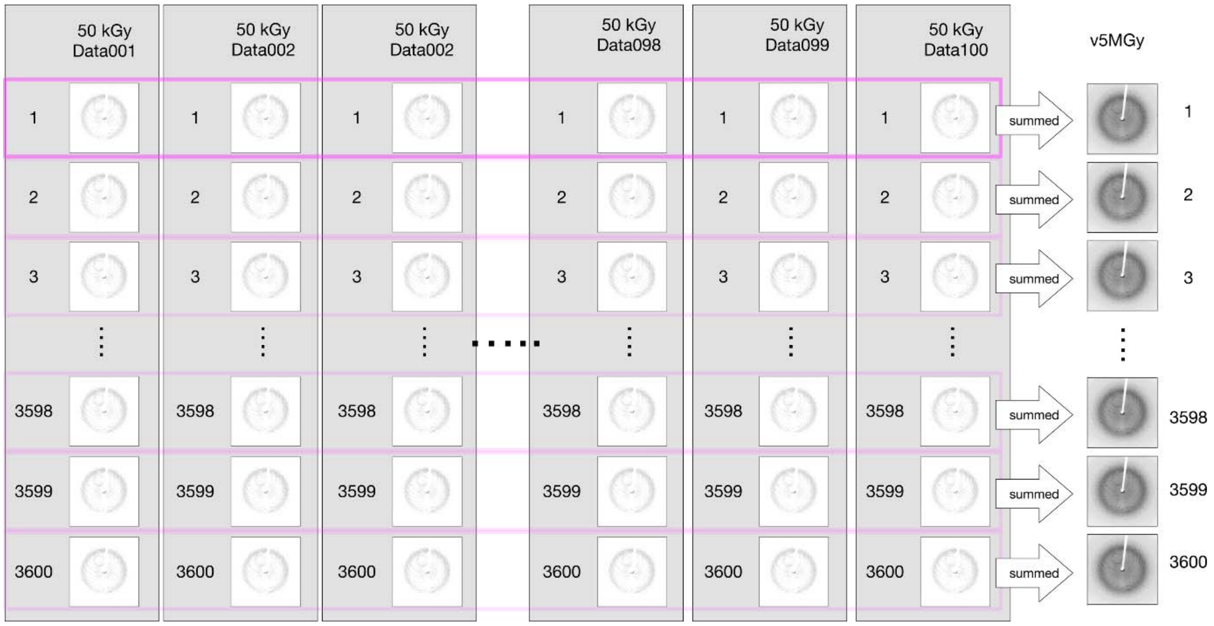


**Figure S1** Summation of 100 sets of 50 kGy data. The v5MGy dataset was synthesized by summing 100 frames of 50kGy data with the same frame number. Each 50 kGy data contains 3,600 frames with 0.1º oscillation width. This process creates a v5.0MGy dataset with frame numbers 1-3600. A similar process would produce 50 sets of 100 kGy data after summing two sets each.

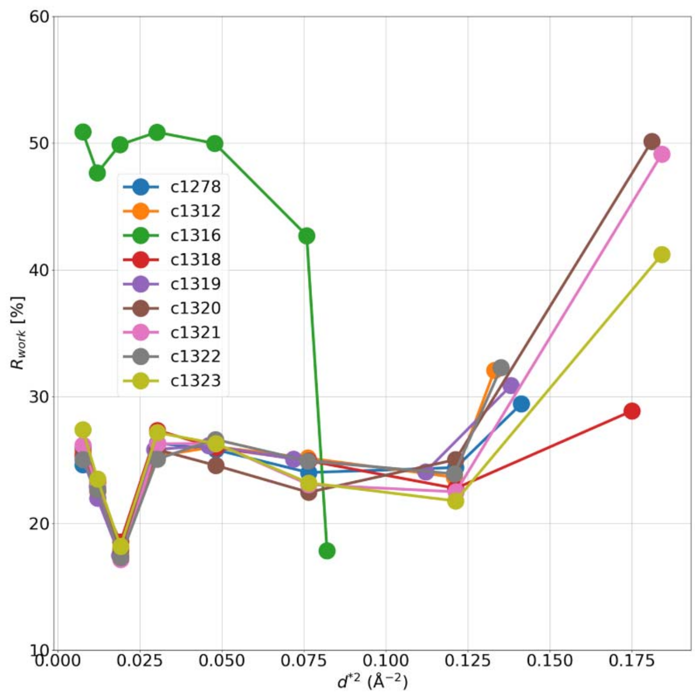


**Figure S2** The *d*-dependency of refinement $R_{\text{work}}$ for representative clusters of CNNM/CorC.

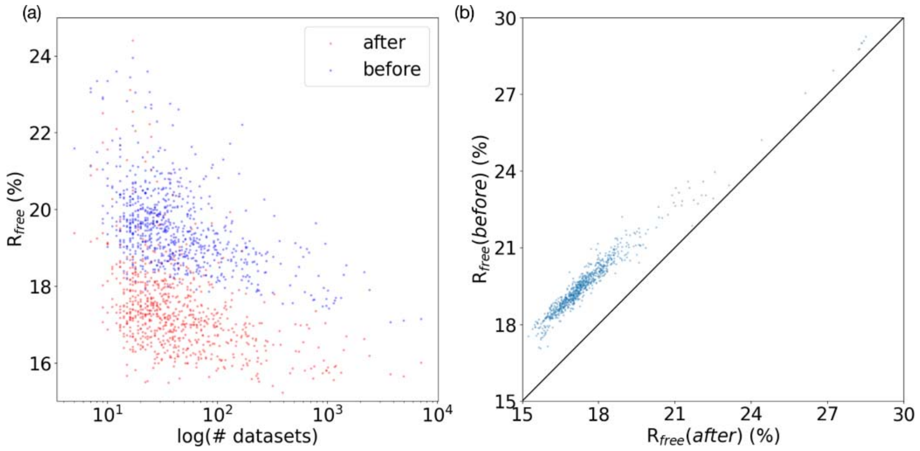


**Figure S3** $R_{\text{free}}$ behaviors of before and after B-blurred refinement of PhC subclusters of C11523. (a) $R_{\text{free}}$ of each cluster dataset is plotted. Blue and red plots represent 'before' and 'after' B-blurred refinement. (b) Relationship between $R_{\text{free}}$ values before (horizontal) and after (vertical) the correction. The black solid line represents the equality line.

**Table S1** Crystallographic data statistics of $AT_2R$

Intensity and refinement statistics of datasets potentially representing polymorphic structures of $AT_2R$ at λ=1.0 Å. (X-ray source: BL32XU, beam size: 10(H)×10(V) µm², oscillation per frame: 0.1°, total wedge: 2–5°)

| | C3203 | C3219 | C3241 | C3244 | C3245 | C3251 | C3252 |
|---|---|---|---|---|---|---|---|
| **Data collection** | | | | | | | |
| Space group | $C222_1$ | $C222_1$ | $C222_1$ | $C222_1$ | $C222_1$ | $C222_1$ | $C222_1$ |
| Cell dimensions | | | | | | | |
| *a*, *b*, *c* (Å) | 104.47, 435.33, 53.55 | 104.52, 434.28, 53.58 | 105.91, 440.66, 54.6, | 104.08, 435.12, 53.64, | 104.31, 432.12, 53.62 | 104.2, 434.02, 53.54 | 104.36, 433.82, 53.58 |
| $\alpha=\beta=\gamma$ (°) | 90 | 90 | 90 | 90 | 90 | 90 | 90 |
| Resolution (Å) | 50.0-3.86 (4.00-3.86) | 50.0-3.77 (3.90-3.77) | 50.0-10.0 (10.36-10.00) | 50.0-3.88 (4.02-3.88) | 50-4.05 (4.19-4.05) | 50-3.56 (3.69-3.56) | 50-3.26 (3.38-3.26) |
| $R_{\text{pim}}$ (%) | 18.6 (36.6) | 20.4 (93.0) | 30.5 (40.0) | 22.9 (62.1) | 38.3 (101.5) | 17.4 (113.0) | 18.9 (105.0) |
| $CC_{1/2}$ | 94.8 (46.6) | 95.2 (52.3) | 79.9 (30.1) | 92.4 (52.3) | 86.7 (55.1) | 97.4 (52.0) | 91.1 (50.1) |
| $\langle I / \sigma I \rangle$ | 10.2 (1.7) | 9.3 (1.2) | 1.5 (1.1) | 8.3 (1.4) | 7.8 (1.6) | 10.2 (1.1) | 16.5 (1.2) |
| Completeness (%) | 99.8 (100.0) | 99.2 (99.0) | 95.5 (100.0) | 95.7 (98.5) | 99.7 (100.0) | 99.8 (100.0) | 99.2 (100.0) |
| Redundancy | 46.5 (44.9) | 40.22 (39.2) | 5.20 (5.74) | 32.1 (31.0) | 27.0 (28.5) | 60.5 (56.2) | 285.0 (292.9) |
| **Refinement** | | | | | | | |
| Resolution (Å) | 25.0-3.86 | 25.0-3.77 | 25.0-10.0 | 25.0-3.88 | 25.0-4.05 | 25.0-3.56 | 25.0-3.26 |
| No. reflections | 12,034 | 12,812 | 788 | 11,354 | 10,379 | 15,159 | 19,551 |
| $R_{\text{work}}$ / $R_{\text{free}}$ (%) | 24.1/29.6 | 25.1/31.1 | 35.0/45.1 | 24.1/31.0 | 24.9/30.6 | 24.8/30.4 | 24.8/30.6 |

| | | | | | | | |
|---|---|---|---|---|---|---|---|
| No. atoms | | | | | | | |
| Protein | 6,283 | 6,283 | 6,283 | 6,283 | 6,283 | 6,283 | 6,283 |
| *B*-factors (Å$^2$) | | | | | | | |
| Protein | 72.3 | 75.0 | 124.7 | 70.6 | 81.3 | 84.3 | 81.3 |
| R.m.s. deviations | | | | | | | |
| Bond lengths (Å) | 0.010 | 0.010 | 0.014 | 0.010 | 0.010 | 0.010 | 0.010 |
| Bond angles (°) | 1.847 | 2.011 | 2.853 | 1.928 | 1.876 | 1.987 | 2.106 |

**Table S2** Crystallographic data statistics of CNNM/CorC

Intensity and refinement statistics of datasets potentially representing polymorphic structures of CNNM/CorC for λ=0.9790 Å. (X-ray source: BL32XU, Beamsize: 10(H) × 15(V) $\mu m^2$, oscillation / frame: 0.1° and Total wedge: 10°)

| | C1278 | C1312 | C1316 | C1318 | C1319 |
|---|---|---|---|---|---|
| **Data collection** | | | | | |
| Space group | *C*222 | *C*222 | *C*222 | *C*222 | *C*222 |
| Cell dimensions | | | | | |
| *a*, *b*, *c* (Å) | 57.46, 83.28, 98.61 | 57.45, 83.28, 98.67 | 57.48, 83.25, 98.74 | 57.45, 83.28, 98.61 | 57.4, 83.35, 98.6 |
| $\alpha$, $\beta$, $\gamma$ (°) | 90 | 90 | 90 | 90 | 90 |
| Resolution (Å) | 49.30-2.66 (2.77-2.66) | 47.29-2.74 (2.84-2.74) | 49.37 - 3.49 (3.61- 3.49) | 49.30 - 2.39 (2.48- 2.39) | 49.30-2.69 (2.79-2.69) |
| $R_{pim}$ (%) | 9.7 (1,060) | 13.0 (1,400) | 23.0 (84.7) | 11.5 (-0.525) | 16.5 (-2.704) |
| $CC_{1/2}$ | 99.5 (49.9) | 99.2 (58.6) | 92.7 (41.6) | 99.7 (49.8) | 98.7 (53.3) |
| $\langle I / \sigma I \rangle$ | 10.5 (0.9) | 9.3 (0.8) | 5.1 (1.1) | 14.0 (0.3) | 8.2 (0.6) |
| Completeness (%) | 99.7 (100.0) | 99.8 (100.0) | 97.8 (100.0) | 99.7 (99.89) | 99.74 (100.0) |
| Redundancy | 62.1 (61.6) | 64.3 (64.2) | 25.3 (25.7) | 167.8 (138.8) | 61.2 (59.9) |
| **Refinement** | | | | | |
| Resolution (Å) | 25.0-2.66 | 25.0-2.74 | 25.0-3.49 | 25.0-2.39 | 25.0-2.69 |
| No. reflections | 7,057 | 6,479 | 3,148 | 9,402 | 6,755 |
| $R_{work}$ / $R_{free}$ (%) | 0.246/0.272 | 0.248/0.275 | 0.443/0.551 | 0.250/0.260 | 0.252/0.278 |

| | | | | | |
|---|---|---|---|---|---|
| No. atoms | | | | | |
| Protein | 2,419 | 2,419 | 2,419 | 2,419 | 2,419 |
| *B*-factors (Å$^2$) | | | | | |
| Protein | 53.3 | 53.8 | 37.6 | 50.5 | 49.5 |
| R.m.s. deviations | | | | | |
| Bond lengths (Å) | 0.011 | 0.011 | 0.010 | 0.010 | 0.011 |
| Bond angles (°) | 2.075 | 2.003 | 2.080 | 1.810 | 2.024 |

**Table S3** Crystallographic data statistics of PhC

Intensity and refinement statistics of datasets potentially representing polymorphic structures of PhC for λ=1.0 Å. (X-ray source: BL32XU, Beamsize: 5(H) × 5(V) μm$^2$, oscillation / frame: 0.1° and Total wedge: 10°)

| | C11523 | C11524 |
|---|---|---|
| **Data collection** | | |
| Space group | *I*23 | *I*23 |
| Cell dimensions | | |
| $a = b = c$ (Å) | 103.69 | 103.83 |
| $\alpha = \beta = \gamma$ (°) | 90.0 | 90.0 |
| Resolution (Å) | 25.0-1.07 | 25-1.21 |
| $R_{\mathrm{pim}}$ (%) | 1.8 (0.2) | 7.6 (2.0) |
| $CC_{1/2}$ | 100.0 | 88.6 |
| | (99.2) | (50.1) |
| $<I / \sigma I>$ | 70.3 | 10.9 |
| | (2.1) | (1.3) |
| Completeness (%) | 100.0 | 99.9 |
| | (100.0) | (99.7) |
| Redundancy | 5638.9 | 2879.3 |
| | (614.7) | (1508.7) |
| | | |
| **Refinement** | | |
| Resolution (Å) | 25.0-1.07 | 25.0-1.20 |
| No. reflections | 71,160 | 53,455 |
| $R_{\mathrm{work}} / R_{\mathrm{free}}$ (%) | 14.9/16.0 | 27.3/27.2 |
| No. atoms | | |
| Protein | 3,908 | 3,908 |
| Water | 545 | 445 |
| *B*-factors (Å$^2$) | | |
| Protein | 13.3 | 11.7 |

| | | |
|---|---|---|
| Water | 19.5 | 16.7 |
| R.m.s. deviations | | |
| Bond lengths (Å) | 0.012 | 0.011 |
| Bond angles (°) | 1.903 | 1.905 |

**Table S4** Crystallographic data statistics of Thermolysin

| | r5.0MGy | v5.0MGy | 50kGy | v100kGy | v250kGy | v500kGy | v1.0MGy |
|---|---|---|---|---|---|---|---|
| **Data collection** | | | | | | | |
| Space group | $P6_122$ | $P6_122$ | $P6_122$ | $P6_122$ | $P6_122$ | $P6_122$ | $P6_122$ |
| Cell dimensions | | | | | | | |
| $a$=$b$, $c$ (Å) | 92.50, 130.56 | 92.37, 130.36 | 92.30, 130.37 | 92.35, 130.42 | 92.37, 130.43 | 92.40, 130.44 | 92.41, 130.45 |
| $\alpha$=$\beta$, $\gamma$ (°) | 90, 120 | 90, 120 | 90, 120 | 90, 120 | 90, 120 | 90, 120 | 90, 120 |
| Resolution (Å) | 50.0-1.50 | 50.0-1.50 | 50.0-1.80 | 50.0-1.64 | 50-1.50 | 50-1.55 | 50-1.50 |
| | (1.55-1.50) | (1.55-1.50) | (1.86-1.80) | (1.70-1.64) | (1.55-1.50) | (1.61-1.55) | (1.55-1.50) |
| $R_{pim}$ (%) | 4.8 | 5.6 | 2.6 | 3.2 | 4.4 | 4.2 | 4.9 |
| | (69.4) | (72.7) | (18.1) | (39.3) | (73.9) | (16.4) | (77.2) |
| $CC_{1/2}$ | 99.9 | 99.9 | 99.9 | 99.9 | 99.9 | 99.9 | 99.9 |
| | (45.2) | (33.3) | (85.5) | (66.2) | (33.2) | (50.5) | (41.6) |
| $<I / \sigma I>$ | 10.4 | 10.8 | 19.3 | 17.8 | 14.9 | 16.0 | 13.9 |
| | (1.1) | (1.0) | (0.0) | (1.0) | (1.2) | (1.4) | (1.1) |
| Completeness (%) | 100.0 | 100.0 | 98.2 | 100.0 | 100.0 | 100.0 | 100.0 |
| | (100.0) | (100.0) | (87.9) | (100.0) | (100.0) | (99.9) | (100.0) |
| Redundancy | 39.0 | 38.7 | 3967.3 | 1961.4 | 784.3 | 391.1 | 195.6 |
| | (39.5) | (39.4) | (4031.0) | (1786.3) | (794.0) | (391.5) | (198.2) |
| **Refinement** | | | | | | | |
| Resolution (Å) | 25.0-1.50 | 25.0-1.50 | 25.0-1.80 | 25-1.64 | 25-1.50 | 25-1.55 | 25-1.50 |
| No. reflections | 52 698 | 49 001 | 28 132 | 40 833 | 49 697 | 48 257 | 51 203 |
| $R_{work}$ / $R_{free}$ (%) | 20.8/22.6 | 20.8/22.9 | 31.2/36.8 | 19.2/20.8 | 20.1/22.5 | 20.5/23.8 | 20.6/22.7 |
| No. atoms | | | | | | | |
| Protein | 4710 | 4710 | 4710 | 4710 | 4710 | 4710 | 4710 |
| Water | 348 | 338 | 149 | 363 | 337 | 346 | 365 |
| $B$-factors (Å$^2$) | | | | | | | |

| | | | | | | | |
|---|---|---|---|---|---|---|---|
| Protein | 17.4 | 15.7 | 31.3 | 20.4 | 16.6 | 17.1 | 16.5 |
| Water | 32.2 | 29.8 | 34.3 | 33.4 | 31.5 | 31.2 | 30.5 |
| R.m.s. deviations | | | | | | | |
| Bond lengths (Å) | 0.006 | 0.005 | 0.008 | 0.006 | 0.006 | 0.006 | 0.006 |
| Bond angles (°) | 0.816 | 0.800 | 0.928 | 0.803 | 0.823 | 0.818 | 0.813 |
